# The Balance of ${\text{HCO}}_{3}^{-}$ Secretion vs. Reabsorption in the Endometrial Epithelium Regulates Uterine Fluid pH

**DOI:** 10.3389/fphys.2018.00012

**Published:** 2018-01-25

**Authors:** Zhang-Dong Xie, Yi-Min Guo, Mei-Juan Ren, Jichun Yang, Shao-Fang Wang, Tong-Hui Xu, Li-Ming Chen, Ying Liu

**Affiliations:** ^1^Key Laboratory of Molecular Biophysics of Ministry of Education, Department of Biophysics and Molecular Physiology, School of Life Science and Technology, Huazhong University of Science and Technology, Wuhan, China; ^2^Department of Physiology and Pathophysiology, School of Basic Medical Sciences, Peking University Health Science Center, Beijing, China; ^3^Wuhan National Laboratory for Optoelectronics, Britton Chance Center for Biomedical Photonics, Huazhong University of Science and Technology, Wuhan, China

**Keywords:** HCO^-^_3_ transporter, HCO^-^_3_ reabsorption, pH regulation, transepithelial ion transport, ion secretion, ion reabsorption, endometrial epithelium

## Abstract

Uterine fluid contains a high concentration of ${\text{HCO}}_{3}^{-}$ which plays an essential role in sperm capacitation and fertilization. In addition, the ${\text{HCO}}_{3}^{-}$ concentration in uterine fluid changes periodically during the estrous cycle. It is well-known that the endometrial epithelium contains machineries involving the apical SLC26 family anion exchangers for secreting ${\text{HCO}}_{3}^{-}$ into the uterine fluid. In the present study, we find for the first time that the electroneutral Na^+^/${\text{HCO}}_{3}^{-}$ cotransporter NBCn1 is expressed at the apical membrane of the endometrial epithelium. The protein abundance of the apical NBCn1 and that of the apical SLC26A4 and SLC26A6 are reciprocally regulated during the estrous cycle in the uterus. NBCn1 is most abundant at diestrus, whereas SLC26A4/A6 are most abundant at proestrus/estrus. In the ovariectomized mice, the expression of uterine NBCn1 is inhibited by β-estradiol, but stimulated by progesterone, whereas that of uterine SLC26A4/A6 is stimulated by β-estradiol. *In vivo* perfusion studies show that the endometrial epithelium is capable of both secreting and reabsorbing ${\text{HCO}}_{3}^{-}$. Moreover, the activity for ${\text{HCO}}_{3}^{-}$ secretion by the endometrial epithelium is significantly higher at estrus than it is at diestrus. The opposite is true for ${\text{HCO}}_{3}^{-}$ reabsorption. We conclude that the endometrial epithelium simultaneously contains the activity for ${\text{HCO}}_{3}^{-}$ secretion involving the apical SLC26A4/A6 and the activity for ${\text{HCO}}_{3}^{-}$ reabsorption involving the apical NBCn1, and that the acid-base homeostasis in the uterine fluid is regulated by the finely-tuned balance of the two activities.

## Introduction

In mammals, acid-base homeostasis in the reproductive tract is critically important for reproduction. The acid-base balance in the reproductive tract influences a series of processes, such as spermatogenesis, sperm capacitation, fertilization, early-stage development and implantation of embryos (for review, see Pastor-Soler et al., 2005; Chan et al., 2007; Liu et al., 2012; Chan and Sun, 2014). While rendered quiescent in the acidic environment of the cauda epididymis of the male reproductive tract where the ${\text{HCO}}_{3}^{-}$ concentration ([${\text{HCO}}_{3}^{-}$]) is very low (Levine and Marsh, 1971), the spermatozoa, upon ejaculation, must undergo a capacitation prior to fertilizing the eggs in the female reproductive tract. It has been well recognized for over half a century that uterine fluid can stimulate the capacitation of spermatozoa (Vishwakarma, 1962; Murdoch and White, 1968). It is now clear that ${\text{HCO}}_{3}^{-}$ is one of the key factors in the uterine fluid that influences sperm capacitation. Indeed, ${\text{HCO}}_{3}^{-}$ is indispensable for the activation of the soluble adenylyl cyclase (sAC) that plays an essential role for sperm capacitation and fertilization (Chen et al., 2000; Hess et al., 2005; for review, see Buffone et al., 2014). Not surprisingly, the [${\text{HCO}}_{3}^{-}$] in the uterine fluid is usually much higher than that in the blood plasma (for review, see Chan et al., 2007; Liu et al., 2012; Chan and Sun, 2014).

The molecular mechanisms underlying ${\text{HCO}}_{3}^{-}$ secretion by the endometrial epithelium have been extensively studied during the past decades. The secretion of ${\text{HCO}}_{3}^{-}$ involves a series of membrane transporters and channels expressed in the endometrial epithelium (for review, see Chan et al., 2007; Liu et al., 2012; Chan and Sun, 2014). At the apical membrane of the endometrial epithelium, members of the solute carrier family 26 (SLC26), such as SLC26A4 and SLC26A6 (Suzuki et al., 2002; Gholami et al., 2013; Chinigarzadeh et al., 2014), are responsible for secreting ${\text{HCO}}_{3}^{-}$ into the lumen in exchange of the luminal Cl^−^, which is in turn secreted via the cystic fibrosis transmembrane conductance regulator (CFTR). SLC26A3 is also expressed in human endometrial epithelium although its subcellular localization remains to be addressed (Chan and Sun, 2014). The electrogenic Na^+^/${\text{HCO}}_{3}^{-}$ cotransporter NBCe1 (SLC4A4) in the basolateral membrane of endometrial epithelium likely contributes to the secretion of ${\text{HCO}}_{3}^{-}$ by mediating ${\text{HCO}}_{3}^{-}$ uptake from the interstitial fluid (Wang et al., 2002; Gholami et al., 2014; for review, see Liu et al., 2012).

On the other hand, it is characteristic of the physiology of the female reproductive tract that the volume and electrolyte composition of the uterine fluid undergo periodical changes during the estrous cycle (or menstrual cycle in human). The volume of the uterine fluid is greatly expanded at proestrus/estrus but becomes much smaller at diestrus (Clemetson et al., 1977). The increase in the fluid volume would cause a distension of the uterus, favoring the swimming and delivery of capacitated spermatozoa to the oviductal tube. In contrast, the decrease in the uterine fluid volume during diestrus stage would cause a closure of the uterus, favoring the implantation of embryo (for review, see Chan et al., 2007). Notably, the [${\text{HCO}}_{3}^{-}$] in the oviductal and uterine fluid is substantially higher at estrus stage when the luminal fluid volume is large than that at diestrus when the luminal fluid volume is small (Vishwakarma, 1962; Maas et al., 1977; Mannowetz et al., 2011).

A question that arises is whether the endometrial epithelium contains a reabsorptive activity for ${\text{HCO}}_{3}^{-}$ that lowers the luminal [${\text{HCO}}_{3}^{-}$] of the uterine fluid during the transition from estrus to diestrus. If so, what is the molecular mechanism responsible for the reabsorption of ${\text{HCO}}_{3}^{-}$? How is its expression regulated during the estrous cycle? The present study was designed to address these questions. In a previous study, Liu et al. have shown by cDNA cloning that *Slc4a7* encoding the electroneutral Na^+^/${\text{HCO}}_{3}^{-}$ cotransporter NBCn1 is expressed in mouse uterus (Liu et al., 2013a). Hereafter, the term “*Slc4a7*” is used when referring to the gene, whereas “NBCn1” is used when referring to its protein product. In the present study, we find that NBCn1 is localized at the apical membrane of the endometrial epithelium in the uteri of mouse and rat. Moreover, we find that the expression of NBCn1 and that of SLC26A4/A6 in the uterus are reciprocally regulated during the estrous cycle in mouse. *In vivo* perfusion studies provide evidence for the presence of ${\text{HCO}}_{3}^{-}$ reabsorption activity in the endometrial epithelium of rat uterus. Our data show that, the endometrial epithelium contains both a ${\text{HCO}}_{3}^{-}$ secretion pathway which is well established, and a ${\text{HCO}}_{3}^{-}$ reabsorption pathway which is novel in the present study. Our study indicate that the [${\text{HCO}}_{3}^{-}$] of uterine fluid is finely controlled by the balance of endometrial ${\text{HCO}}_{3}^{-}$secretion and reabsorption.

## Materials and methods

### Animals

All protocols for animal care and usage were approved by the Institutional Research Ethics Committee at Huazhong University of Science and Technology (Wuhan, China). Adult C57/BL mice and adult Sprague-Dawley (SD) rats were purchased from the Hubei Research Center of Experimental Animals (Wuhan, Hubei, China). The animals were housed in standard rodent cages with free access to rodent chow and tap water. All animals were aged 9–11 weeks when used for experiments in the present study.

Typically, the estrous cycle of adult mice and rats is divided into proestrus, estrus, metestrus, and diestrus. In our present study, the estrous stages of adult mice and rats were determined by cytological evaluation of vaginal smears as described previously (Byers et al., 2012). Briefly, the vaginal smear contained the mixture of leukocytes and nucleated epithelial cells at proestrus stage, predominately cornified epithelial cells at estrus stage, the mixture of leukocytes and cornified epithelial cells at metestrus stage, and predominately leukocytes at diestrus stage. Vaginal smear was sampled at least twice a day from each animal. The animal was evaluated for at least one full estrous cycle before it was used for further experiments. The duration of a full estrous cycle was ~4 days for the mice and rats.

### Antibodies

Normal rabbit IgG was purchased from Beyotime (Shanghai, China). Normal goat IgG was purchased from Santa Cruz Biotechnology (Santa Cruz, California, USA). Primary antibodies and secondary antibodies used in the present study are summarized in the Tables 1, 2, respectively.

**Table 1:** Primary antibodies.

**Antibody**	**Species**	**Source**	**Cat#**	**Dilution**
Anti-NBCn1	Rabbit	Abcam	ab82335	1:5,000 (WB), 1:50 (IF)
Anti-NBCn2	Rabbit	custom-made		1:20 (IF)
Anti-SLC26A4	Rabbit	Santa Cruz	sc-50346	1:2,000 (WB), 1:20 (IF)
Anti-SLC26A6	Goat	Abcam	ab156935	1:2,000 (WB), 1:20 (IF)

**Table 2:** Secondary antibodies.

**Antibody**	**Conjugate**	**Source**	**Cat#**	**Dilution**
Goat-anti-rabbit	HRP	Beyotime	A0208	1:10,000 (WB)
Goat-anti-mouse	HRP	Beyotime	A0216	1:10,000 (WB)
Donkey-anti-goat	HRP	ProteinTech	SA00001-3	1:10,000 (WB)
Donkey-anti-goat	Alexa 488	Jackson	705-545-003	1:100 (IF)
Goat-anti-rabbit	Dylight 549	EarthOx	E032320-01	1:100 (IF)

### Cloning of NBCn1 cDNA from rat uterus

Total RNA was prepared from the uteri of adult rats with TRIzol® reagent (Life Technologies Corporation, Carlsbad, CA, USA) according to the instructions of the manufacturer. Single-stranded complementary DNA (cDNA) was synthesized with reverse transcriptase of Moloney Murine Leukemia Virus (M-MLV; Life Technologies). The cDNA encoding full-length NBCn1 was then amplified by nested polymerase chain reactions (PCR) with primer pairs rNBCn1-GSP-F1 (5′-actactcccgggCGTCCTCTGGCTCTCTCAGTCCTC-3′) plus rNBCn1-GSP-R1 (5′-GGTTGATATGATTGATTGCCACTGACAGAG-3′) for first round PCR and rNBCn1-GSP-F1 plus rNBCn1-GSP-R2 (5′-actactgcggccgcTGGTGCTCACAACAAACATCTGATGCTAC-3′) for second round PCR, respectively. The DNA products were restricted with XmaI and NotI, subcloned into pGH19 vector, and transformed into bacteria for identification of NBCn1 variants. Single colonies were sequenced for full-length to verify the specific details of NBCn1 variants.

### Heterologous expression of NBCn1 and NBCn2 in xenopus oocytes

*Xenopus* oocytes were prepared as described previously (Liu et al., 2013a). Briefly, a *Xenopus* laevis was anesthetized with 0.2% ethyl-3-aminobenzoate methanesulfonate (Sigma-Aldrich, MO, USA). An ovary lobe was collected, cut into small pieces, and digested with 2 mg/ml collagenase (Sigma-Aldrich) for 90 min at room temperature. The oocytes were then washed 5 times with Ca^2+^-free NRS solution (in mM: 82 NaCl, 2 KCl, 20 MgCl_2_, 5 Hepes; pH 7.50; 200 mOsm) and 5 times with ND96 (96 NaCl, 2 KCl, 1 MgCl_2_, 1.8 CaCl_2_, 5Hepes;pH7.50; 200 mOsm). Oocytes at stages V-VI were selected and incubated in OR3 medium at 18°C.

The plasmids containing cDNA encoding mouse NBCn1 (accession# JQ073566) tagged with EGFP at its amino terminus or rat NBCn2 (accession# JX073717) tagged with EGFP at its carboxyl terminus have been described previously (Liu et al., 2013a,b). The plasmids were linearized at the 3' untranslated region by restriction with NotI (Thermo Fisher Scientific, MA, USA). cRNAs were prepared with mMESSAGE mMACHINE® kits (Thermo Fisher Scientific) according to instructions of the manufacturer. 50 nl of cRNA (0.5 ng/nl) was injected into each oocyte. Control oocyte was injected with 50 nl of H_2_O. The oocytes were incubated in OR3 medium for 4 days and then collected for membrane protein preparations.

### Membrane protein preparation and western blot analysis

For tissue collection, the animals were anesthetized by subcutaneous injection of pentobarbital sodium. The tissues were frozen in liquid nitrogen immediately upon removal from the animal and then stored at −80°C until usage. For membrane protein preparation, a tissue was placed in a straight glass tube containing protein isolation buffer (in mM: 7.5 NaH_2_PO_4_, 250 sucrose, 5 EDTA, 5 EGTA, pH 7.4) plus 1% protease inhibitor cocktail (cat#P7340, Sigma-Aldrich Inc., St. Louis, MO, USA) and homogenized by a PTFE pestle on a Glas-Col High Speed Homogenizer (Glas-Col LLC., Terre Haute, IN, USA). The crude homogenate was centrifuged at 3,000 *g* for 10 min at 4°C to remove cell debris. The supernatant was ultracentrifuged at 100,000 *g* for 1 h at 4°C. The resultant pellet was collected and resuspended in a buffer (in mM: 20 Tris-HCl pH 7.5, 5 EDTA pH 8.0) containing 5% sodium dodecyl sulfate (SDS). Protein concentration was determined by using enhanced BCA protein assay kit (Beyotime, Shanghai, China). The membrane preparations were then stored in aliquots at −80°C until usage.

For western blotting, the membrane proteins were separated by 8% SDS-polyacrylamide gel electrophoresis (SDS-PAGE) and then blotted onto a PVDF membrane (Millipore, Bedford, MA, USA). The membrane was blocked with 5% milk in 1 × TBST (in mM: 1 Tris, 150 NaCl, 0.1% Tween20, pH 7.4) for 2 h at room temperature (RT), and then incubated with primary antibody overnight at 4°C. After 5 × 6 min washes with 1 × TBST, the membrane was incubated with HRP-conjugated secondary antibody at RT for 2 h followed by 5 × 6 min washes with 1 × TBST. Chemiluminescense was performed with SuperSignal® West Pico Chemiluminescent Substrate (Thermo Scientific, Rockford, IL, USA) and detected with an X-ray film. Densitometry analysis was performed with ImageJ, a free image processing software from NIH.

### Immunofluorescence

The mouse or rat was transcardially perfused with phosphate buffered saline (PBS; in mM: 77.4 Na_2_HPO_4_, 22.6 NaH_2_PO_4_, pH7.4) containing heparin (10 unit/mL), and then with PBS containing 4% paraformaldehyde (PFA). The tissues were embedded in OCT medium, and frozen sections of 10 μm were prepared and stored at −20°C until usage. The section was baked at 60°C overnight on a heating block, rehydrated in 1 × TBS (in mM: 1 Tris, 150 NaCl, pH 7.4) for 1 h, and washed with 1 × TBST for 5 × 6 min. The section was incubated in buffer containing 2% sodium citrate at 95°C for 20 min for antigen retrieval. The section was then blocked with 5% Normal Goat Serum (NGS) in 1 × TBS at RT for 30 min, incubated with primary antibody in 1 × TBS containing 2.5% NGS and 0.025% TritonX-100 overnight at 4°C. The section was then washed with 1 × TBST for 5 × 6 min, incubated with secondary antibody 1 × TBS containing 2.5% NGS and 0.025% TritonX-100 at RT for 1 h, washed for 3 × 6 min with 1 × TBS, counter-stained with 4,6-diamidino-2-phenylindole dihydrochloride (DAPI; Beyotime) at RT for 5 min, washed with 1 × TBS for 3 × 6 min, and mounted in PVP mounting medium. The images were acquired on a FV1000 confocal laser scanning microscope (Olympus, Shinjuku, Japan).

### Ovariectomization and hormone treatment

Adult C57BL/6J mice (8 weeks old, body weight ~23 g) were anesthetized by intraperitoneal injection of chloral hydrate/xylazine (dose: 400/10 mg per kg body weight) and bilaterally ovariectomized. Two weeks after the surgery, each mouse was subcutaneously injected with either 100 ng of β-estradiol (cat#E2758, Sigma) in 0.2 ml sesame oil (cat#S3547, Sigma, MO, USA), or 1 mg of progesterone (cat#P8783, Sigma) in 0.2 ml sesame oil. The control was injected with just 0.2 ml of sesame oil only. Two injections were applied with an interval of 24 h. The uteri were collected 12 h after the second injection.

### *In vivo* perfusion of uterus

For *in vivo* perfusion, an adult rat was anesthetized with intraperitoneal injection of pentobarbital sodium (80 mg/Kg). Surgery was started when the rat had no response to puncture stimulus. An incision was made on the right side of the abdomen (close to the position of the ovary) to cannulate the perfusion tube at the distal end of the uterus horn. A second incision was made along the midline of the abdomen to cannulate the collecting tube at the proximal end of the uterus horn. The perfusion tube was connected to a syringe pump (RWD202, RWD Life Science Co., Ltd., Shenzhen, China) for the delivery of perfusate.

Prior to initiation of the perfusion, a given volume (360 μl) of initial perfusate was introduced into the perfusion tube. Both sides of this perfusate were flanked with an air bubble of ~50 μl. The perfusate was then delivered into the uterus by the syringe pump at a constant rate of 1.5 μl·min^−1^. The perfusion was terminated when the perfusate between the two air bubbles was completely flushed out and collected. The volume of this “collected effluent fluid” was quantified. The concentration of total CO_2_, the concentrations of the major electrolytes, and pH of the collected fluid were determined by using a PL2100 Blood-Gas Analyzer (Perlong Medical Equipment Co., Ltd., Nanjing, China). The average length of the uteri used for perfusion was 4.01 ± 0.06 cm and was not significantly different between the rats at estrus and diestrus. During the entire perfusion process, additional pentobarbital sodium (10 mg per Kg body weight) was injected every 2 h to keep the rat unconscious. The rat was sacrificed by cervical dislocation after the perfusion.

**5% CO_2_/50 mM ${\text{HCO}}_{3}^{-}$ Ringer's solution:** (in mM) 76 NaCl, 15 KCl, 1 MgCl_2_, 10 glucose, 5 HEPES, adjusting to pH 7.6. The solution was supplemented with 50 mM NaHCO_3_, and then bubbled with 5% CO_2_ (balanced with N_2_). The pH of the final solution was 7.6.

**Nominally ${\text{HCO}}_{3}^{-}$-free HEPES buffer:** (in mM) 126 NaCl, 15 KCl, 1 MgCl_2_, 10 glucose, 5 HEPES, adjust pH to 7.4.

### Data analysis

Data are presented as mean ± SEM (standard error of mean). One-way ANOVA followed by Fisher's *post-hoc* multiple comparisons was performed for statistical analysis with Minitab® 16 (Minitab Inc., State College, PA, USA). Two-tailed Student's *t*-test was performed for statistical analysis on the perfusion data. *p* < 0.05 was considered statistically significant.

## Results

### Expression of NBCn1, SLC26A4, and SLC26A6 in rodent uterus

**Figure 1:**
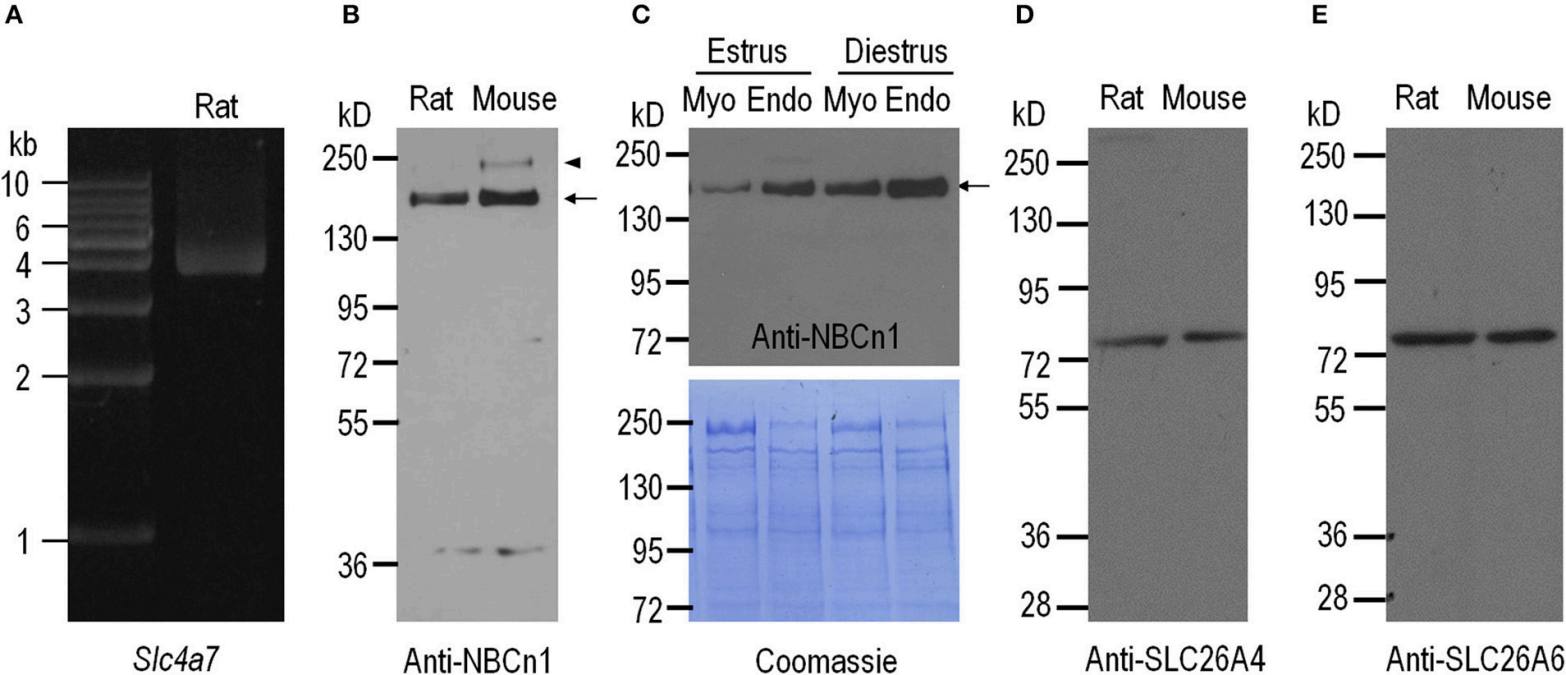
Expression of NBCn1, SLC26A4, and SLC26A6 in uteri of adult rat and mouse. **(A)** RT-PCR analysis for expression of *Slc4a7* encoding NBCn1 in the uterus of adult rat. **(B)** Western blotting analysis for expression of NBCn1 in the uteri of adult rat and mouse. **(C)** Western blotting analysis for expression of NBCn1 in endometrial layer (Endo) and myometrial layer (Myo) of rat uteri at estrus and diestrus (upper panel) and Coomassie Brilliant Blue staining of the blot (lower panel). **(D,E)** Western blotting analysis for expression of SLC26A4 and SLC26A6 in the uteri of adult rat and mouse. For quantitative comparison, 20 μg of total membrane proteins were loaded for each lane in **C**. The arrows in **B,C** indicate the presumable glycosylated monomer of NBCn1, whereas the arrowhead in **B** indicates the presumable dimer of NBCn1. NBCn1 dimer was also observed in rat uterus upon a longer exposure (data not shown). Each panel is representative of three independent experiments.

The *Slc4a7* gene encoding NBCn1 contains two alternative promoters, the distal promoter P1 and the proximal promoter P2. Promoter P1 gives rise to the expression of a group of NBCn1 variants starting with “MEAD,” whereas P2 gives rise to the expression of three groups of NBCn1 variants, each starting with “MERF,” “MIPL,” and “MDEL,” respectively (Pushkin et al., 1999; Choi et al., 2000; Liu et al., 2013a; Wang et al., 2015). In a previous study, Liu et al. have shown that expressed in mouse uterus are three MEAD-NBCn1 variants i.e., NBCn1-E, -G, and -I (for details of NBCn1 variants; see Liu et al., 2013a) derived from the distal promoter P1, but not the variants derived from P2. In the present study, we obtained, by RT-PCR, a product encoding MEAD-NBCn1 from rat uterus (Figure 1A). The PCR product was subcloned into a cloning vector for identification of NBCn1 variants. By full-length sequencing, we identified three different NBCn1 variants: NBCn1-C (accession# AAF14345), -G (accession# KP721461), and -I (accession# KP721460). Consistent with the previous study by Liu et al. (2013a), in the present study, we were not able to detect, by RT-PCR, the expression of the NBCn1 (e.g., MERF-NBCn1) derived from the proximal promoter P2 of *Slc4a7* in rat uterus. Taken together, it appears that the expression of NBCn1 in the uterus is specifically controlled by promoter P1 of *Slc4a7*. The expression of multiple variants indicates that NBCn1 likely plays a complicated role in the uterus.

We employed western blotting to examine the expression of NBCn1 in the uteri of mouse and rat. The specificity of anti-NBCn1 was validated by western blotting with NBCn1 heterologously expressed in *Xenopus* oocytes (see Supplementary Figure S1). As shown in Figure 1B, anti-NBCn1 recognizes a major band with a molecular weight (MW) of ~150 kD (indicated by arrow) and an additional band with higher MW (arrowhead in Figure 1B) from the uteri of mouse and rat. This 150-kD band and the higher band presumably represent the glycosylated monomer and dimer of NBCn1, respectively (Chen et al., 2007). Western blotting with finely dissected tissues showed that NBCn1 expression is more abundant in the endometrial layer than it is in the myometrial layer of the uterus, for both estrus and diestrus (Figure 1C). Moreover, NBCn1 expression is more abundant in the tissues at diestrus compared to the corresponding tissues at estrus. It is interesting that, a previous study showed that the activity of *Slc4a7* promoter is present in the myometrium layer, but not detectable in the endometrium layer of mouse uterus, as determined by using LacZ as a reporter (Boedtkjer et al., 2008). A likely explanation for the apparent inconsistency between our antibody data and the previous LacZ data is the difference in the genomic background between the wild-type mice and the LacZ-containing ones.

We then examined, by western blotting, the expression of SLC26A4 and SLC26A6 in the uteri of mouse and rat. Anti-SLC26A4 recognizes a single band with an apparent MW of ~78 kD in the uteri of rat and mouse (Figure 1D). Similarly, anti-SLC26A6 recognizes a band with an apparent MW of ~78 kD in the uteri of rat and mouse (Figure 1E), consistent with the previous observations (He et al., 2010).

### Cellular localization of NBCn1, SLC26A4, and SLC26A6 in rodent uterus

**Figure 2:**
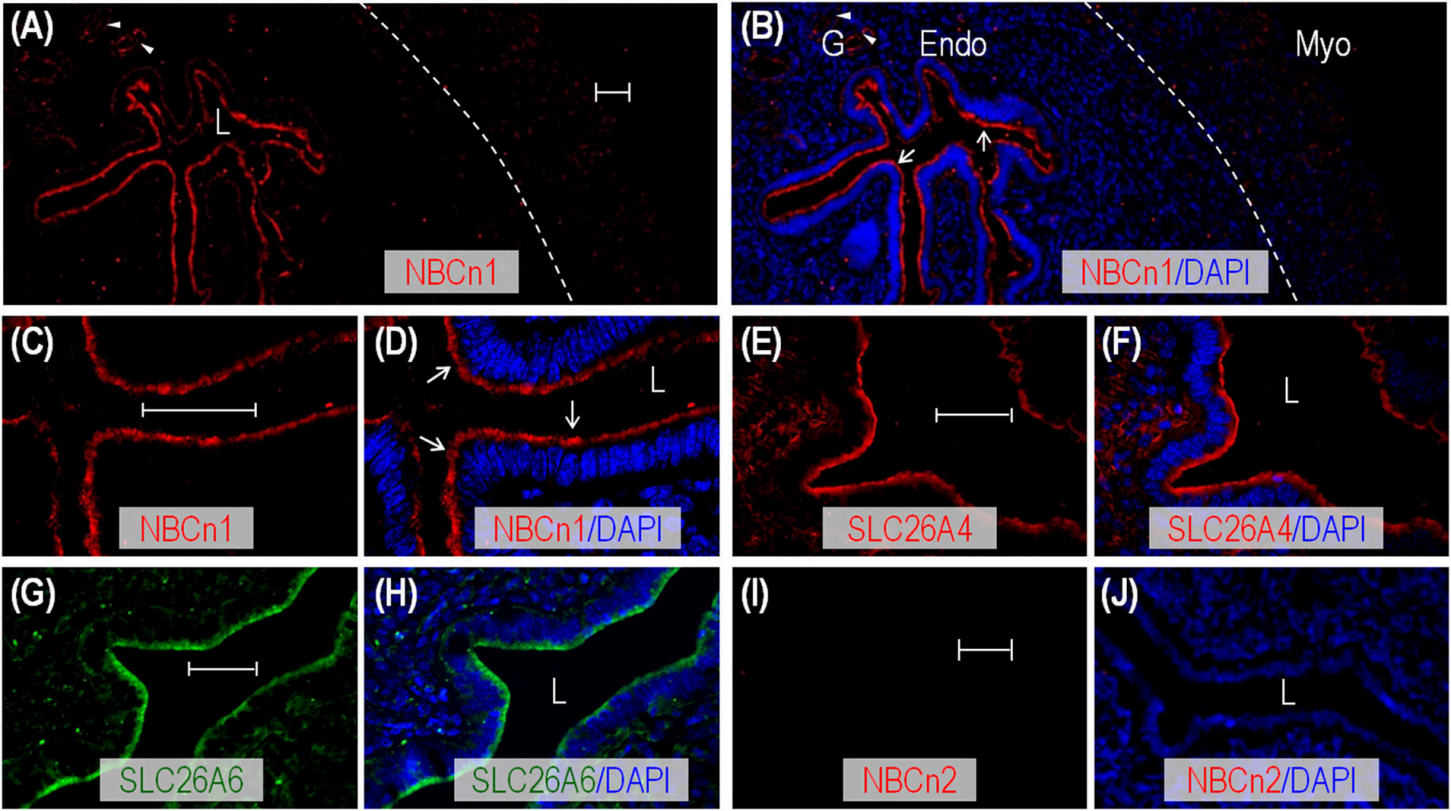
Indirect immunofluorescence of NBCn1, SLC26A4, and SLC26A6 in uterus of adult mouse. **(A,B)** Overview of NBCn1 expression **(A)** and its merge with DAPI **(B)** in a section of mouse uterus. **(C,D)** High magnification view showing apical localization of NBCn1 in endometrial epithelium in section of mouse uterus. **(E,F)** Apical localization of SLC26A4 in a section of mouse uterus. **(G,H)** Apical localization of SLC26A6 in a section of mouse uterus. **(I,J)** Lack of staining by anti-NBCn2 in section of mouse uterus. In these experiments, the mice were not intentionally examined to determine their estrous stages when sacrificed for uterus collection. The dashed line in **B** indicates the demarcation between the myometrium layer (Myo) and endometrium layer (Endo). L: uterus lumen; G: glandular duct. Arrows indicate NBCn1 expression at the apical membrane of endometrial epithelium. Arrowheads indicate NBCn1 expression at the apical membrane of glandular epithelium. Scale bars: 40 μm. Results are representative of 3–4 independent experiments with sections from two different mice.

We employed indirect immunofluorescence to examine the tissue and subcellular localization of NBCn1, SLC26A4, and SLC26A6 in the uteri of mouse and rat. An overview shows that the fluorescence signal derived from anti-NBCn1 is highly enriched in the endometrial epithelia in the sections of mouse uterus (arrows in Figures 2A,B and Supplementary Figure S2), an observation in line with Figure 1C. NBCn1 is also expressed to a lesser extent in the glandular epithelia (arrowheads in Figures 2A,B and Supplementary Figure S2). A high magnification view shows that NBCn1 is predominantly expressed at the apical membrane of the endometrial epithelium in the uterus (Figures 2C,D). Consistent with previous studies (Suzuki et al., 2002; Gholami et al., 2013; Chinigarzadeh et al., 2014), our data show that SLC26A4 (Figures 2E,F) and SLC26A6 (Figures 2G,H) are predominantly expressed at the apical membrane of endometrial epithelia in mouse uterus. No apparent fluorescence signal was observed in immunofluorescence study on uterus sections by using anti-NBCn2 directed against NBCn2—a close homolog of NBCn1 in the SLC4 family (Figures 2I,J), normal IgG, or by omitting the primary antibodies (data not shown).

Similar to the cases in mouse, by indirect immunofluorescence, we found that NBCn1, SLC26A4, and SLC26A6 are also predominantly expressed at the apical membrane of the endometrial epithelium (indicated by arrows) in the sections of rat uterus (Supplementary Figures S3A–F). NBCn1 and SLC26A4 are also expressed at the apical membrane of structures that presumably represent the glandular ducts (arrowheads in Supplementary Figures S3A–F).

The apical localization of NBCn1 is of particular interest. Under physiological conditions, the electroneutral Na^+^/${\text{HCO}}_{3}^{-}$ cotransporter NBCn1 localized at the apical membrane would mediate ${\text{HCO}}_{3}^{-}$ influx from the lumen into the endometrial epithelium driven by the inwardly-directed electrochemical gradient of Na^+^. Our data suggest that the endometrial epithelium contains a ${\text{HCO}}_{3}^{-}$ reabsorption pathway.

### Estrous-cycle-dependent expression of NBCn1, SLC26A4, and SLC26A6 in mouse uterus

**Figure 3:**
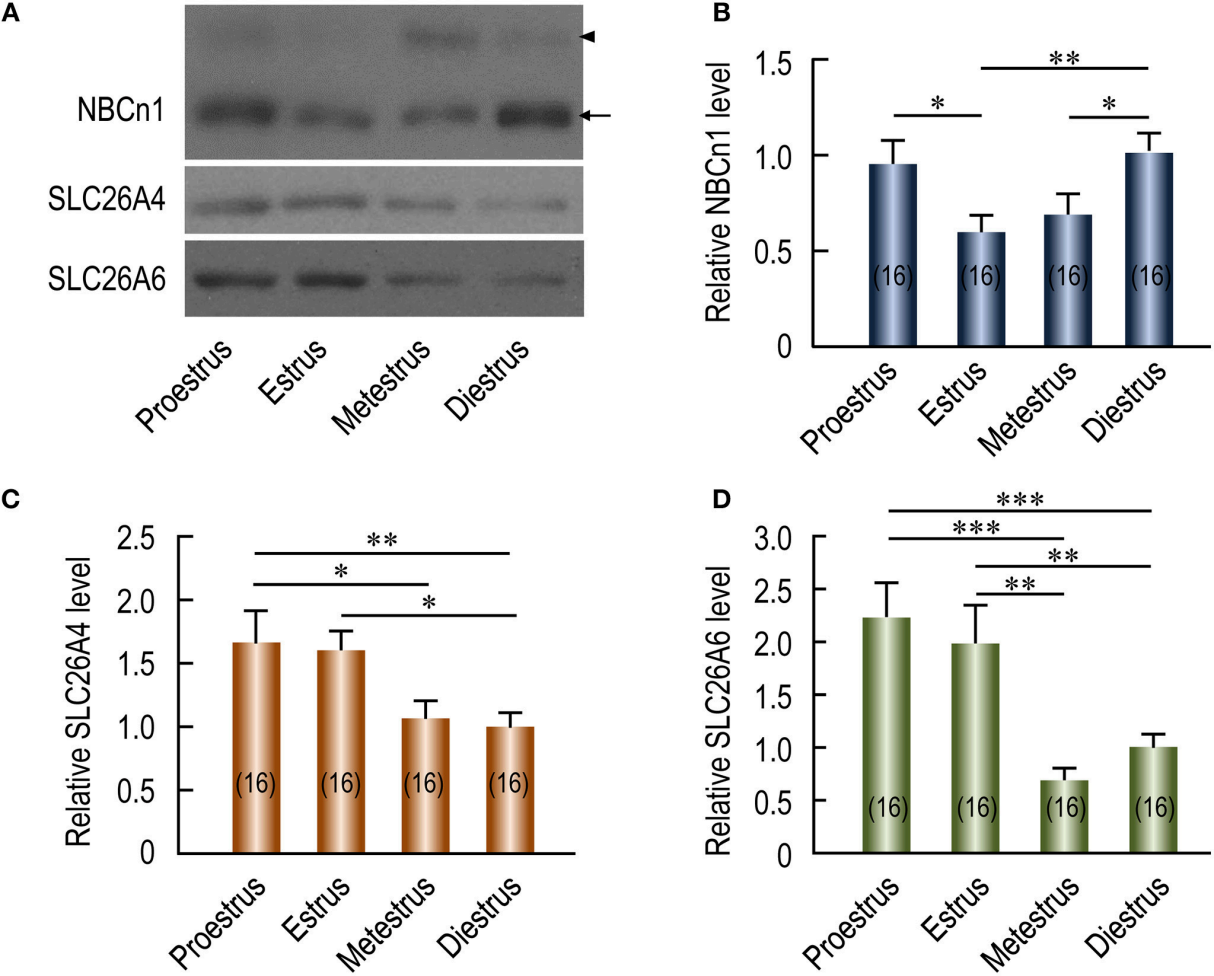
Effect of estrous stages on expression of NBCn1, SLC26A4, and SLC26A6 in mouse uteri. **(A)** Representative western blotting of NBCn1, SLC26A4, and SLC26A6 in mouse uteri at different estrous stages. **(B,D)** Summary showing the relative abundance of uterine NBCn1 **(B)**, SLC26A4 **(C)**, and SLC26A6 **(D)** in mouse uteri at different estrous stages. In **A**, each lane represents the uterus from a single mouse. Equal amount (10 μg) of total membrane proteins were loaded into each lane on the same gel for western blotting analysis. Equal loading was verified by Coomassie staining (see details in Supplementary Figures S4D–F). Full-length blots for the images in **A** are shown in Supplementary Figures S4A–C. To create the bar graphs, raw densitometric density was obtained for the target band in each lane on a specific blot by using ImageJ. The density of the “diestrus” lanes on this blot was averaged. The raw densitometric density of each individual lane was then divided by this average density of the “diestrus” lanes to create a normalized value, representing the relative protein abundance of the transporter in the membrane preparation. Such normalized values from different blots were pooled to create the bars in **B–D**. The data were presented as mean ± SEM. The figures in the parenthesis of each bar represent the number of the mice included in the corresponding group, each mouse analyzed individually. One-way ANOVA followed by Fisher's *post-hoc* multiple comparisons was performed for statistical analysis. ^*^p < 0.05; ^**^p < 0.01; ^***^p < 0.001.

The expression of ${\text{HCO}}_{3}^{-}$ transporters in the plasma membrane of endometrial epithelium involved in the regulation of uterine fluid environment could cyclically change during the estrous cycles. If NBCn1 is involved in ${\text{HCO}}_{3}^{-}$ reabsorption in the endometrial epithelium, we would expect that the regulation of the expression of uterine NBCn1 during the estrous cycle differs from that of the SLC26A4 and SLC26A6 that are involved in ${\text{HCO}}_{3}^{-}$ secretion by the endometrial epithelium. To examine the changes in the expression level of these ${\text{HCO}}_{3}^{-}$ transporters during the estrous cycle, crude membrane preparations from the uteri of mice at different estrous stages were separated by SDS-PAGE for western blotting analysis. Figure 3A shows the representative results of western blotting for NBCn1, SLC26A4, and SLC26A6 (see full blots in Supplementary Figures S4A–C). No systemic bias was observed in the overall loading of total proteins in the lanes for different estrous stages as verified by Coomassie Brilliant Blue staining (for example, see Supplementary Figures S4D–F). The expression of β-actin in the membrane preparations of mouse uteri at proestrus, estrus, and diestrus is not significantly different from each other, but is significantly lower than that at metestrus (Supplementary Figure S5), suggesting that the expression of β-actin is not well conserved throughout the entire estrous cycle. As summarized in Figure 3B, the relative abundance of NBCn1 is highest at diestrus and falls from proestrus to metestrus. In contrast, the relative abundance of SLC26A4 (Figure 3C) and SLC26A6 (Figure 3D) is highest at proestrus/estrus, and falls greatly during metestrus and diestrus.

**Figure 4:**
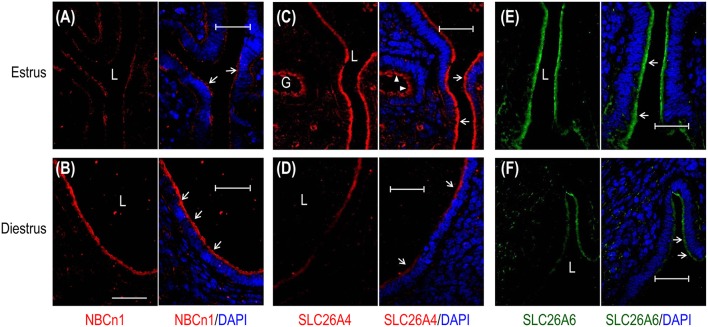
Comparison of cellular expression of ${\text{HCO}}_{3}^{-}$ transporters in mouse uteri at estrus vs. diestrus by indirect immunofluorescence. **(A,B)** NBCn1 in the uterus at estrus and diestrus. **(C,D)** SLC26A4 in the uterus at estrus and diestrus. **(E,F)** SLC26A6 in the uterus at estrus and diestrus. In these experiments, the sections were prepared from the uteri of mice that were sacrificed at typical estrus or diestrus stages as determined by cytological evaluation of the vaginal smears. To compare the expression of a given transporter at estrus vs. diestrus, immunofluorescence staining was performed in parallel with the sections of estrus and diestrus, and images were acquired with the same set of parameters on the confocal microscopy. L: uterus lumen; G: glandular duct. Arrows indicate the apical membrane of endometrial epithelium. Arrowheads indicate the apical membrane of glandular epithelium. Scale bars: 40 μm. Results are representative of 3–4 independent experiments for each transporter with sections from one mouse at either estrus or diestrus.

We then examined, by indirect immunofluorescence, the cellular expression of the ${\text{HCO}}_{3}^{-}$ transporters in mouse uteri at estrus vs. diestrus. NBCn1 is expressed at the apical membrane of endometrial epithelium at both estrus (Figure 4A) and diestrus (Figure 4B). It appears that NBCn1 is also slightly expressed at the basolateral membrane of the endometrial epithelium at estrus stage (Figure 4A). Note that, the fluorescence intensity of the apical NBCn1 in the endometrial epithelium at estrus is lower than that at diestrus, suggesting that the expression of apical NBCn1 in the endometrial epithelium is up-regulated during the transition from estrus to diestrus. In contrast, the expression of the apical SLC26A4 in the endometrial epithelium at estrus (Figure 4C) is higher than that at diestrus (Figure 4D). The same is true for SLC26A6 (Figures 4E,F). The last two lines of observations suggest that the expression of the apical SLC26A4 and SLC26A6 in the endometrial epithelium is down-regulated during the transition from estrus to diestrus. Finally, the observations from the indirect immunofluorescence are consistent with the western blotting data shown in Figure 3.

Taken the western blotting data and the immunofluorescence data together, NBCn1 exhibits an expression profile distinct from those of the SLC26A4 and SLC26A6 in mouse uteri during the estrous cycle. Our data suggest that NBCn1 plays a physiological role distinct from what SLC26A4 and SLC26A6 do in the uterus.

### Expressional regulation of uterine NBCn1, SLC26A4, and SLC26A6 by steroid hormones in ovariectomized mice

**Figure 5:**
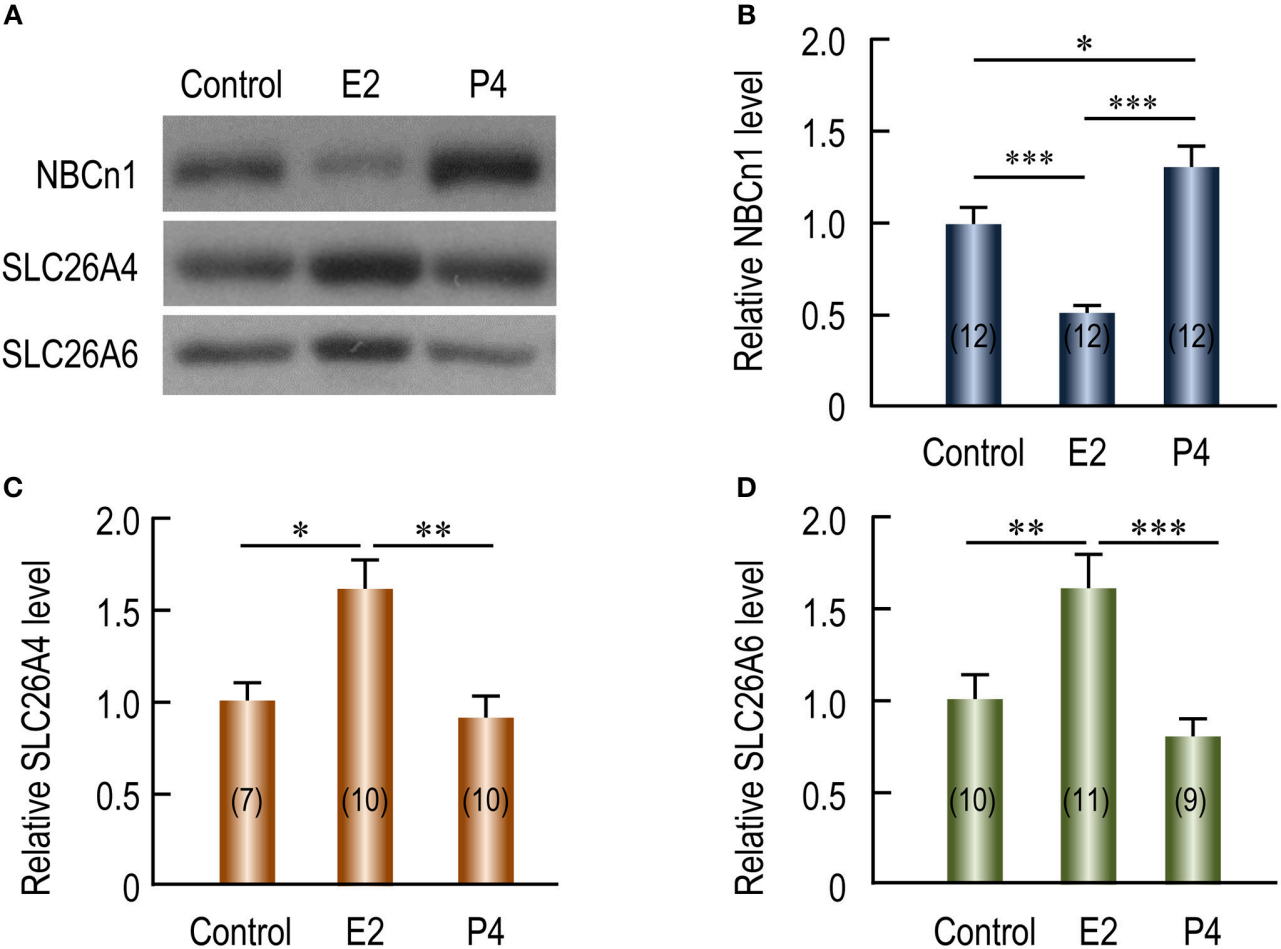
Effects of β-estradiol and progesterone on relative protein abundance of NBCn1, SLC26A4, and SLC26A6 in uteri of ovariectomized mice. **(A)** Representative western blottings of NBCn1, SLC26A4, and SLC26A6. The ovariectomized mice were injected with sesame oil (control), β-estradiol (E2), or progesterone (P4). Each lane represents the uterus from a single mouse. Equal amount (10 μg) of membrane proteins were loaded into each lane for western blotting. Full-length blots for the images in **A** are shown in Supplementary Figures S4A–C. Equal loading was verified by Coomassie staining (see Supplementary Figures S4D). **(B–D)** Summary showing the effects of E2 and P4 on relative abundance of uterine NBCn1 **(B)**, SLC26A4 **(C)**, and SLC26A6 **(D)**. The bar graphs were created by using a strategy similar to that in Figure 3. Briefly, raw densitometric density was obtained for the target band in each lane on a specific blot by ImageJ. The raw densitometric densities were then divided by the average density of all “control” lanes on the same blot to create a normalized value, representing the relative protein abundance of the transporter in the membrane preparation. Such normalized values from different blots were pooled to generate the bars in **B–D**. The data were presented as mean ± SEM. The figures in the parenthesis of each bar indicate the number of mice included in each group. One-way ANOVA followed by Fisher's *post-hoc* multiple comparisons was performed for statistical analysis. ^*^p < 0.05; ^**^p < 0.01; ^***^p < 0.001.

The periodical changes in the relative abundances of NBCn1 and SLC26A4/A6 suggest that the expression of these ${\text{HCO}}_{3}^{-}$ transporters is likely regulated by steroid sex hormones. We examined the effects of β-estradiol and progesterone on the expression of the ${\text{HCO}}_{3}^{-}$ transporters in the uteri of ovariectomized mice. Figure 5A shows the representative results of western blotting for NBCn1, SLC26A4, and SLC26A6 with the crude membrane preparations from the uteri of ovariectomized mice. Again, equal loading was verified by Coomassie staining with the blots (Supplementary Figure S6). As summarized in Figure 5B, compared to the control lacking treatment by the hormones, the expression of NBCn1 is significantly decreased by 49% upon the treatment by β-estradiol (*p* < 0.001), and significantly increased by 31% upon the treatment by progesterone (*p* < 0.05). In contrast, compared to the control, upon the treatment by β-estradiol, the expressions of uterine SLC26A4 (Figure 5C) and SLC26A6 (Figure 5D) are significantly increased by 61 and 62%, respectively. The expression of uterine SLC26A4 and SLC26A6 is not significantly affected by progesterone compared to that of the controls. The up-regulation in the expression of uterine SLC26A6 by β-estradiol is consistent with previous observations (He et al., 2010; Chinigarzadeh et al., 2014).

**Figure 6:**
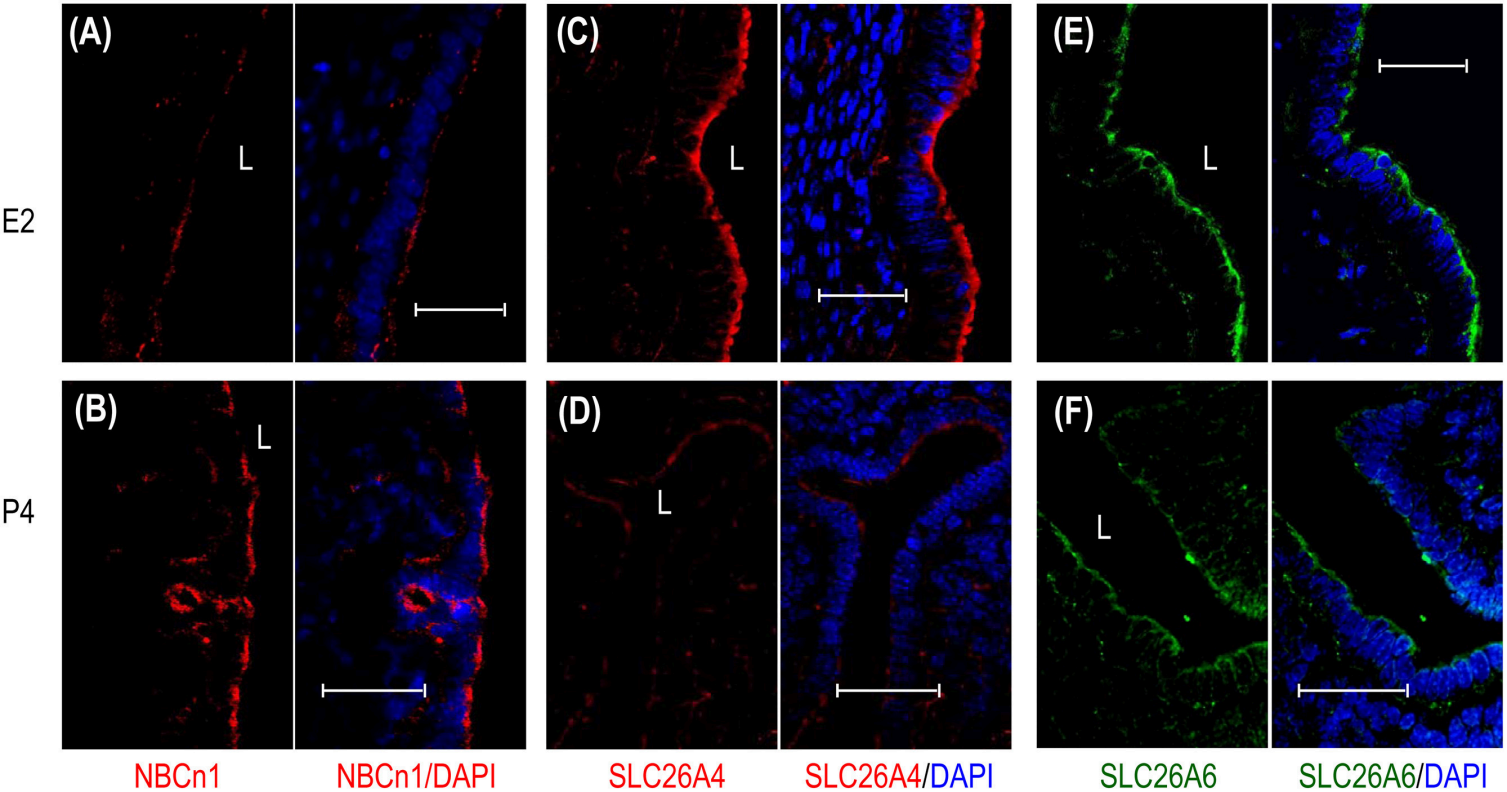
Effect of β-estradiol (E2) and progesterone (P4) on cellular expression of NBCn1, SLC26A4, and SLC26A6 in uteri of ovariectomized mice. **(A,B)** NBCn1 in the uterus from a mouse treated with E2 **(A)** or P4 **(B)**. **(C,D)** SLC26A4 in the uterus treated with E2 **(C)**, or P4 **(D)**. **(E,F)** SLC26A6 in the uterus treated with E2 **(E)** or P4 **(F)**. To compare the effects of E2 vs. P4 on the expression of a given transporter, immunofluorescence staining was always performed in parallel with the sections treated with either E2 or P4, and images were then acquired with the same set of parameters on the confocal microscopy. L: uterus lumen. Scale bars: 40 μm. Results are representative of 3–4 independent experiments with sections from three different mice for each treatment (E2 vs. P4).

By indirect immunofluorescence, we examined the effect of steroid sex hormones on the cellular expression of the ${\text{HCO}}_{3}^{-}$ transporters in the uteri of ovariectomized mice. NBCn1 is localized at the apical membrane of the endometrial epithelium in the uteri of the mice treated with β-estradiol (Figure 6A) or progesterone (Figure 6B). However, the fluorescence intensity of the apical NBCn1 in the endometrial epithelium treated with β-estradiol is lower than that treated with progesterone. In contrast, the fluorescence intensity of the apical SLC26A4 in the endometrial epithelium treated with β-estradiol (Figure 6C) is higher than that treated with progesterone (Figure 6D). The same is true for SLC26A6 (Figure 6E vs. Figure 6F). The observations from the indirect immunofluorescence are consistent with the western blotting data shown in Figure 5.

**Figure 7:**
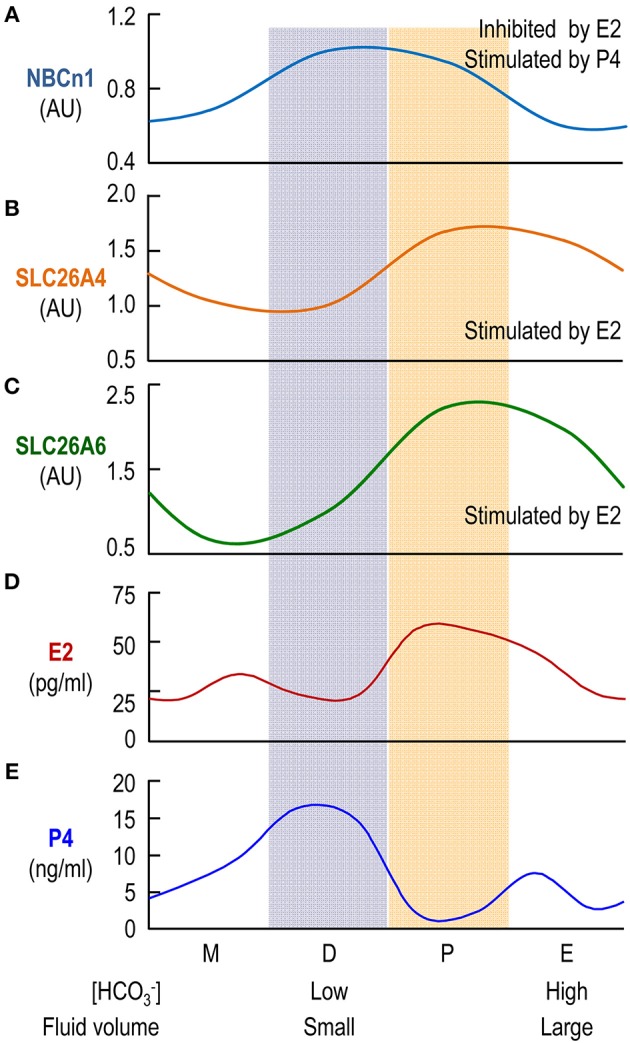
Comparison of profiles of protein abundances of uterine ${\text{HCO}}_{3}^{-}$ transporters and plasma levels of β-estradiol and progesterone. The profiles for the relative protein levels of NBCn1 **(A)**, SLC26A4 **(B)**, SLC26A6 **(C)** are fitted based upon the data shown in Figure 3. The profiles of the blood plasma β-estradiol (E2; **D**) and progesterone (P4; **E**) are plotted based upon the previously reported data (Walmer et al., 1992).

Taken together, our data indicate that the expression of NBCn1 and that of SLC26A4 and SLC26A6 are inversely regulated by the steroid sex hormones. As has shown previously, the β-estradiol level and the progesterone level in blood plasma alternately rise and fall during the estrous cycle (Walmer et al., 1992). In this context, the effect of steroid sex hormones on the expression of NBCn1, SLC26A4 and SLC26A6 can well explain the cyclical changes in the expression of the uterine transporters during the estrous cycle shown in Figure 3 in the present study. Figure 7 shows an alignment of the profiles of the protein abundance of uterine NBCn1 (Figure 7A), SLC26A4 (Figure 7B), and SLC26A6 (Figure 7C) during the estrous cycle from the present study with the profiles of the plasma levels of β-estradiol (Figure 7D) and progesterone (Figure 7E) replotted from the previous study (Walmer et al., 1992). Note that, the profiles of the plasma levels of β-estradiol and progesterone during the estrous cycle are well consistent with the cyclical changes in the relative abundances of uterine NBCn1, SLC26A4, and SLC26A6 given the specific effects of the hormones on the expression of the ${\text{HCO}}_{3}^{-}$ transporters. For example, NBCn1 is most abundant during diestrus (with high level of progesterone and low level of β-estradiol), but is lowest during estrus (with low progesterone and high β-estradiol), consistent with our observations that progesterone is stimulatory, whereas β-estradiol is inhibitory to the expression of NBCn1 in the uterus. Similar analyses are true for SLC26A4 and SLC26A6.

### ${\text{HCO}}_{3}^{-}$ reabsorption of rat uterus by *in vivo* perfusion

**Figure 8:**
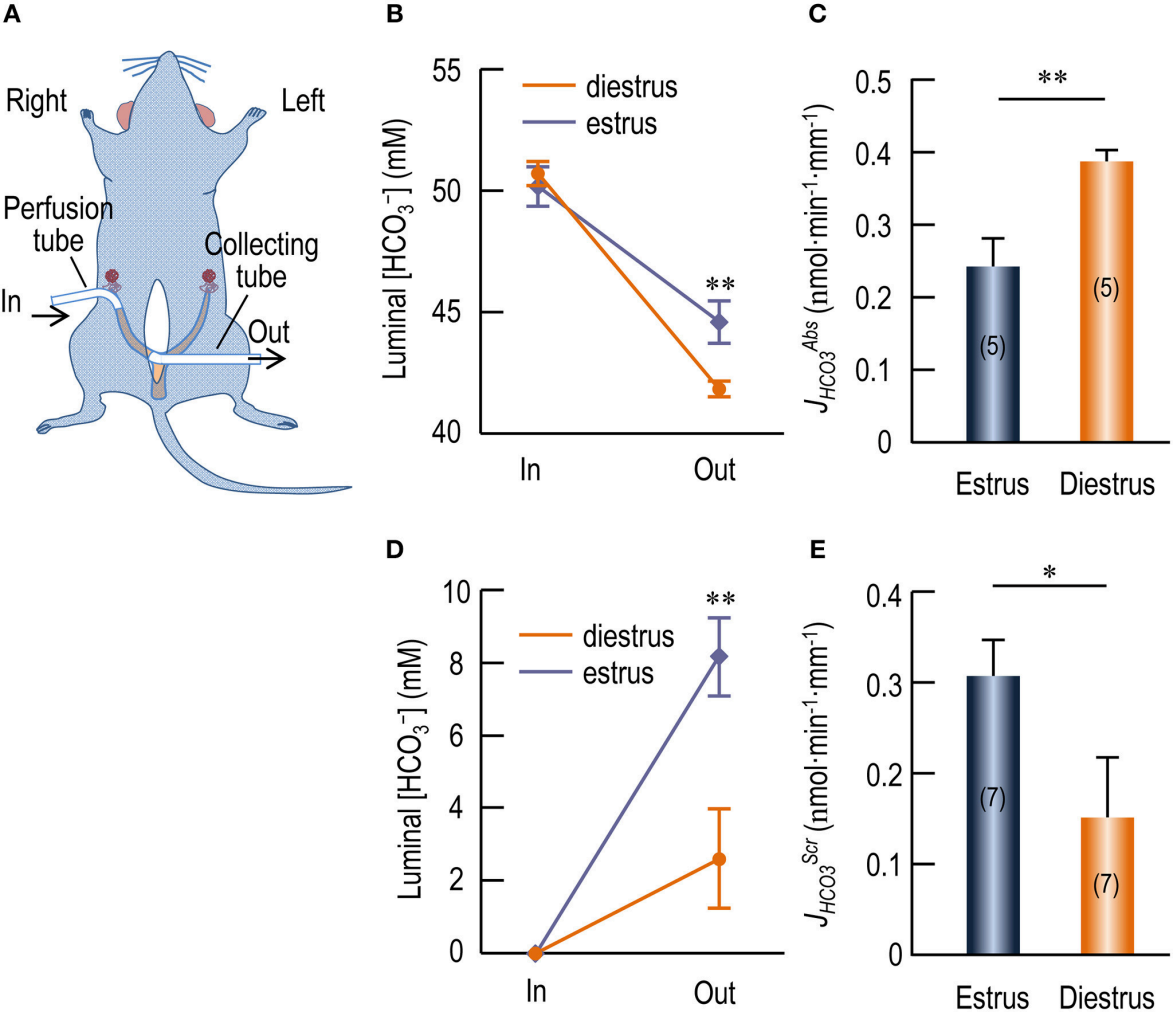
*In vivo* perfusion study showing ${\text{HCO}}_{3}^{-}$ transport by endometrial epithelium of rat uterus. **(A)** Diagram for *in vivo* perfusion of rat uterus. For perfusate delivery, a perfusion tube was cannulated at the distal end of the right uterus horn. For sample collection, a collecting tube was cannulated at the proximal end of the right uterus horn. **(B)** Changes in [${\text{HCO}}_{3}^{-}$] of the luminal perfusate in rat uteri when perfused with Ringers' solution containing 5% CO_2_/50 mM ${\text{HCO}}_{3}^{-}$. **(C)** Rate of ${\text{HCO}}_{3}^{-}$ reabsorption (*J*_HCO3_^*Abs*^) by endometrial epithelium at estrus and diestrus. **(D)** Changes in [${\text{HCO}}_{3}^{-}$] of the luminal perfusate in rat uteri when perfused with nominally ${\text{HCO}}_{3}^{-}$-free HEPES solution. **(E)** Rate of ${\text{HCO}}_{3}^{-}$ secretion (*J*_HCO3_^*Scr*^) by endometrial epithelium at estrus and diestrus. In: Influent fluid; Out: Effluent fluid. Stars in **B,D** indicate significance between estrus and diestrus. Student's *t*-test was performed for statistical comparison between estrus and diestrus. ^*^p < 0.05; ^**^p < 0.01.

We performed *in vivo* perfusion with rat uterus to examine ${\text{HCO}}_{3}^{-}$ transport by the endometrial epithelium. Figure 8A shows a diagram for the *in vivo* perfusion. We perfused the uteri with a Ringer solution containing 5% CO_2_/50 mM ${\text{HCO}}_{3}^{-}$. As shown in Figure 8B, compared to the influent perfusate, the [${\text{HCO}}_{3}^{-}$] in the collected effluent fluid was significantly decreased for both estrus (in mM: 50.16 ± 0.82 in vs. 44.59 ± 0.86 out, *p* < 0.001, *n* = 5) and diestrus (in mM: 50.68 ± 0.49 in vs. 41.82 ± 0.30 out, *p* < 0.001, *n* = 5). Moreover, the [${\text{HCO}}_{3}^{-}$] in the collected effluent fluid for estrus is significantly higher than that for diestrus (*p* < 0.01). The decrease in [${\text{HCO}}_{3}^{-}$] (Δ[${\text{HCO}}_{3}^{-}$]) indicates reabsorption of ${\text{HCO}}_{3}^{-}$ by the endometrial epithelium during the perfusion. Compared to the initial volume (360 μl) introduced into the uteri, the volume of the collected effluent fluid was slightly decreased. However, the volume change (ΔV) was not significantly different between estrus and diestrus (in μl: 44.6 ± 2.8 for estrus vs. 41.8 ± 2.0 for diestrus, *p* = 0.4, *n* = 5). We propose two models for the volume decrease: (a) fluid retention hypothesis; (b) fluid absorption hypothesis.

- **Fluid retention hypothesis:** We assume that the decrease in the volume was derived only from the retention of the perfusate in some poorly flushed spaces in the uterus, e.g., the cavities of the glandular structures. We further assume that the retained fluid had the same [${\text{HCO}}_{3}^{-}$] as the collected effluent fluid did. In this case, we used the following formula for computing $J_{HCO3}^{Abs}$:
  (1) $$J_{HCO3}\;=\;\Delta [{\text{HCO}}_{3}^{-}]\times V_{in}/(t\times L)$$
  where Δ[${\text{HCO}}_{3}^{-}$] represents the difference between the [${\text{HCO}}_{3}^{-}$] (measured as the concentration of total CO_2_) of the original influent perfusate ([${\text{HCO}}_{3}^{-}$]_in_) and that of the collected effluent fluid ([${\text{HCO}}_{3}^{-}$]_out_), *V*_*in*_ represents the volume of the influent fluid, *t* is the duration time of the perfusion for the uterus, and *L* is the length of the uterus for perfusion. The [${\text{HCO}}_{3}^{-}$] of the initial influent perfusate and the collected effluent fluid were pair-measured for each individual perfusion experiment by using the blood gas analyzer. As summarized in Figure 8C, the estimated $J_{HCO3}^{Abs}$ of diestrus is higher by 58% than that of estrus (in nmol·min^−1^·mm^−1^: 0.245 ± 0.038 for estrus vs. 0.389 ± 0.017 for diestrus, *p* = 0.002, *n* = 5 for each).
- **Fluid absorption hypothesis:** We assume that the decrease in the volume was derived only from the absorption of the perfusate by the endometrial epithelia. In this case, we used the following formula (2) for computing $J_{HCO3}^{Abs}$.
  (2) $$J_{HCO3}=({\left[{\text{HCO}}_{3}^{-}\right]}_{\text{in}}\times V_{in}-{\left[{\text{HCO}}_{3}^{-}\right]}_{\text{out}}\times V_{out})/(t\times L)$$
  where *V*_*out*_ is the volume of the collect effluent perfusate, the other parameters have the same meaning as defined for formula (1).

As summarized in Supplementary Figure S7C, the estimated $J_{HCO3}^{Abs}$ of diestrus is again significantly higher by 24% than that of estrus (in nmol·min^−1^·mm^−1^: 0.487 ± 0.026 for estrus vs. 0.602 ± 0.011 for diestrus, *p* = 0.001, *n* = 5 for each).

We presume that both fluid absorption and fluid retention contribute to the volume decrease observed in our experiments. Note that, the above $J_{HCO3}^{Abs}$ calculated based on the fluid retention hypothesis is a minimized estimate for ${\text{HCO}}_{3}^{-}$ absorption by the endometrial epithelium inasmuch as this calculation does not count for the portion of ${\text{HCO}}_{3}^{-}$ that could have been absorbed by the endometrial epithelium. In contrast, the $J_{HCO3}^{Abs}$ calculated based on the fluid absorption hypothesis is a maximized estimate for $J_{HCO3}^{Abs}$. The real $J_{HCO3}^{Abs}$ of the endometrial epithelium would lie somewhere between the two extremities. Thus, our data indicate that the endometrial epithelium at diestrus contains higher activity for ${\text{HCO}}_{3}^{-}$ absorption than the endometrial epithelium at estrus does.

### ${\text{HCO}}_{3}^{-}$ secretion of rat uterus by *in vivo* perfusion

In another set of experiments, we perfused the rat uteri with nominally ${\text{HCO}}_{3}^{-}$-free HEPES solution with no detectable ${\text{HCO}}_{3}^{-}$ by gas analysis. As shown in Figure 8D, compared to the influent perfusate, the [${\text{HCO}}_{3}^{-}$] of the collected effluent fluid was significantly increased for both estrus and diestrus. Moreover, the [${\text{HCO}}_{3}^{-}$] in the collected effluent fluid for estrus is significantly higher than that for diestrus (in mM: 8.16 ± 1.07 for estrus vs. 2.60 ± 1.36 for diestrus, *p* = 0.02, *n* = 7). The accumulation of ${\text{HCO}}_{3}^{-}$ in the luminal fluid indicates ${\text{HCO}}_{3}^{-}$ secretion by the endometrial epithelium during the perfusion. Similar to the perfusion with Ringer solution containing CO_2_/${\text{HCO}}_{3}^{-}$, when perfused with the ${\text{HCO}}_{3}^{-}$-free HEPES solution, the volume of the collected effluent fluid was slightly decreased compared to the volume initially introduced into the uteri. Again, ΔV was not significantly different between estrus and diestrus (in μl: 43.1 ± 2.6 for estrus vs. 48.1 ± 4.3 for diestrus, *p* = 0.3, *n* = 7). Again, we hypothesize that the volume decrease is caused by fluid retention and/or fluid absorption.

Based upon the fluid retention hypothesis, we computed the rate of ${\text{HCO}}_{3}^{-}$ secretion ($J_{HCO3}^{Scr}$) for the perfusion with ${\text{HCO}}_{3}^{-}$-free solution by using the above formula (1). As summarized in Figure 8E, the estimated $J_{HCO3}^{Scr}$ of estrus is higher by 99% than that of diestrus (in nmol·min^−1^·mm^−1^: 0.305 ± 0.040 for estrus vs. 0.153 ± 0.065 for diestrus, *p* = 0.026, *n* = 7 for each).

Based upon the fluid absorption hypothesis, we computed the $J_{HCO3}^{Scr}$ by using the above formula (2). As summarized in Supplementary Figure S7E, the estimated $J_{HCO3}^{Scr}$ of estrus is significantly higher by 119% than that of diestrus (in nmol·min^−1^·mm^−1^: 0.263 ± 0.033 for estrus vs. 0.120 ± 0.047 for diestrus, *p* = 0.01, *n* = 7 for each).

Note that, similar to the analysis for $J_{HCO3}^{Abs}$, the above calculations represent the two extremities of the estimation for $J_{HCO3}^{Scr}$ by the endometrial epithelium at estrus or diestrus. The real $J_{HCO3}^{Scr}$ would lie somewhere between these two values. Thus, our data indicate that the endometrial epithelium at estrus elicits higher ${\text{HCO}}_{3}^{-}$ secretion activity than it does at diestrus.

## Discussion

In the present study, we demonstrate for the first time that the electroneutral Na^+^/${\text{HCO}}_{3}^{-}$ cotransporter NBCn1 is primarily expressed at the apical membrane of the endometrial epithelium in rodent uterus. Moreover, we confirm the apical localization of SLC26A4/A6 in the endometrial epithelium in rodent uterus. Interestingly, the relative protein abundance of uterine NBCn1 and those of uterine SLC26A4/A6 alternately rise and fall during the estrous cycle. Consistently, the protein expression of NBCn1 and those of SLC26A4/A6 are reciprocally regulated by β-estradiol and progesterone in the uteri of ovariectomized mice.

It has been known for a long time that the volume of uterine fluid in the female reproductive tract undergoes periodical change during the estrous cycle, being that the fluid volume is maximized at proestrus/estrus and minimized at diestrus (Shih et al., 1940; Clemetson et al., 1977). The reduction in the uterine fluid volume during the transition from estrus to diestrus is due to fluid reabsorption by the endometrial epithelium rather than fluid leakage via the cervical canal (Clemetson et al., 1977). In rodent uterus, the [${\text{HCO}}_{3}^{-}$] in the uterine fluid at estrus (when fluid volume enlarged) is about twice of the [${\text{HCO}}_{3}^{-}$] in the uterine fluid at diestrus (when fluid volume minimized) (Mannowetz et al., 2011). While the fluid secretion during estrus is accompanied by ${\text{HCO}}_{3}^{-}$ secretion—as has been well recognized, it is reasonable to speculate that fluid reabsorption during the transition from estrus to diestrus is accompanied by ${\text{HCO}}_{3}^{-}$ reabsorption by the endometrial epithelium. In the present study, the apical localization of NBCn1 provides molecular evidence that the endometrial epithelium indeed contains a ${\text{HCO}}_{3}^{-}$ reabsorption pathway. Moreover, our *in vivo* perfusion study provides functional evidence for the presence of ${\text{HCO}}_{3}^{-}$ reabsorption activity in the endometrial epithelium.

It is interesting that the endometrial epithelium simultaneously contains the activities of reabsorption and secretion for ${\text{HCO}}_{3}^{-}$. Our *in vivo* perfusion study indicates that, it depends on the specific [${\text{HCO}}_{3}^{-}$] in the initial perfusate (50 mM vs. null in our cases) whether the endometrial epithelium performs ${\text{HCO}}_{3}^{-}$ reabsorption or secretion in our perfusion experiment. The endometrial epithelium presumably contains a sensor for the luminal pH and [${\text{HCO}}_{3}^{-}$] to regulate the activities for ${\text{HCO}}_{3}^{-}$ reabsorption and secretion to finely control the acid-base homeostasis in the luminal fluid. The threshold of the sensor, which presumably defines the steady-state [${\text{HCO}}_{3}^{-}$] of the luminal fluid, would dynamically change according to specific estrous stages. Our data indicate that the steady-state [${\text{HCO}}_{3}^{-}$] in the uterine fluid at estrus or diestrus likely lies somewhere below 50 mM. Consistent with this hypothesis, the [${\text{HCO}}_{3}^{-}$] of the uterine fluid is reported to be 42.9 mM in superovulated mouse (Mannowetz et al., 2011). It is reasonable to speculate that, if the [${\text{HCO}}_{3}^{-}$] of the Ringer's solution used for the *in vivo* perfusion equals the physiological steady-state [${\text{HCO}}_{3}^{-}$] in the luminal fluid of the uterus, one would see neither absorption nor secretion of ${\text{HCO}}_{3}^{-}$ during the perfusion of the uterus.

**Figure 9:**
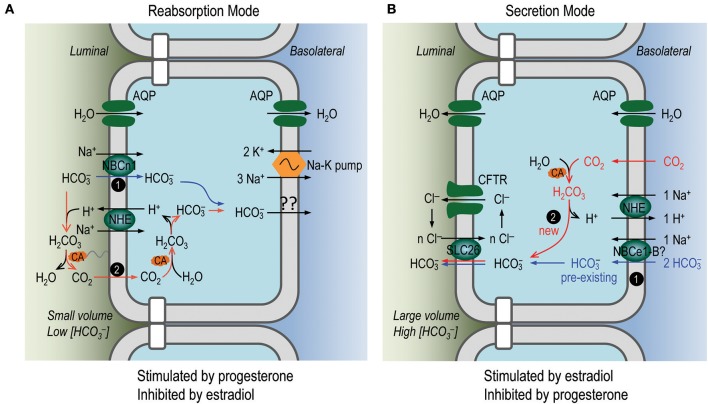
Proposed model for reabsorption and secretion of ${\text{HCO}}_{3}^{-}$ in endometrial epithelium. **(A)** Secretion of ${\text{HCO}}_{3}^{-}$ by the endometrial epithelium. **(B)** Reabsorption of ${\text{HCO}}_{3}^{-}$ by the endometrial epithelium. In the reabsorption mode, the endometrial epithelium would perform net fluid reabsorption, whereas in the secretion mode, the endometrial epithelium would perform net fluid secretion, via members of aquaporins, such as AQP2, AQP3, AQP5 expressed at the apical and/or basolateral membranes of the endometrial epithelium (Mobasheri et al., 2005; Hildenbrand et al., 2006; Skowronski, 2010; for review, see Zhu et al., 2015). Our expression data and *in vivo* perfusion data indicate that the activities for ${\text{HCO}}_{3}^{-}$ reabsorption and secretion are present at the same time in the endometrial epithelium. We propose that the activity for secretion is dominant during estrus stage when the uterine fluid volume is large and [${\text{HCO}}_{3}^{-}$] is high, whereas the activity for reabsorption is dominant during diestrus stage when the uterine fluid volume is small and [${\text{HCO}}_{3}^{-}$] is low.

We attempted to replace the luminal Na^+^ with N-methyl-D-glucamate (NMDG^+^) to test the Na^+^-dependence of the ${\text{HCO}}_{3}^{-}$ absorption. However, NMDG appeared to be toxic to the uterus in our *in vivo* perfusion experiments. Nevertheless, the ${\text{HCO}}_{3}^{-}$ absorption in the perfusion with 5% CO_2_/50 mM ${\text{HCO}}_{3}^{-}$ solution was unlikely to be mediated by the apical anion exchangers such as SLC26A4 and SLC26A6 (see “${\text{HCO}}_{3}^{-}$ flux via the endometrial epithelium” in Supplementary Material). Instead, the ${\text{HCO}}_{3}^{-}$ absorption could be explained by the apical NBCn1 which mediates ${\text{HCO}}_{3}^{-}$ uptake driven by the inwardly-directed electrochemical gradient of Na^+^ (blue pathway #1 in reabsorption mode, Figure 9A). It could also be explained by a mechanism dependent on proton secretion mediated by the apical NHEs (red pathway #2, Figure 9A) that are expressed in the endometrial epithelium (Wang et al., 2003; Chinigarzadeh et al., 2015a). By this pathway, the luminal ${\text{HCO}}_{3}^{-}$ is titrated to form CO_2_ and H_2_O under the influence of membrane-associated carbonic anhydrase (CA). The CO_2_ then diffuses into the endometrial epithelium to re-create ${\text{HCO}}_{3}^{-}$. The mechanism for ${\text{HCO}}_{3}^{-}$ reabsorption at the basolateral membrane of the endometrial epithelium remains to be addressed. At the apical side, it is possible that both NBCn1 and NHEs contribute to the ${\text{HCO}}_{3}^{-}$ absorption observed in our *in vivo* perfusion study. However, further functional studies are necessary to characterize the specific role of NBCn1 vs. NHEs in the reabsorption of the luminal ${\text{HCO}}_{3}^{-}$ in uterine fluid by the endometrial epithelium.

In the case of nominally ${\text{HCO}}_{3}^{-}$-free solution, the ${\text{HCO}}_{3}^{-}$ secretion by the endometrial epithelium would be largely attributable to the anion exchangers such as SLC26A4 and SLC26A6, although one could not completely rule out a contribution by NBCn1 (see “${\text{HCO}}_{3}^{-}$ flux via the endometrial epithelium” in Supplementary Material). The apical SLC26 transporters could be fueled by two sources of intracellular ${\text{HCO}}_{3}^{-}$: (1) the pre-existing ${\text{HCO}}_{3}^{-}$ uptaken by the basolateral Na^+^/${\text{HCO}}_{3}^{-}$ cotransporters, such as NBCe1 (Fong et al., 1998; Wang et al., 2002; Gholami et al., 2014) (blue pathway #1 in secretion mode, Figure 9B); (2) the new ${\text{HCO}}_{3}^{-}$ created from CO_2_ under the influence of CA (red pathway #2 in Figure 9B). The proton generated during the creation of this new ${\text{HCO}}_{3}^{-}$ could be extruded by the basolateral NHE (Wang et al., 2003).

The reabsorption mode is likely stimulated by progesterone and inhibited by β-estradiol. Indeed, progesterone has stimulatory effect on the fluid reabsorption by the endometrial epithelium (Naftalin et al., 2002; Salleh et al., 2005). The epithelial Na^+^ channel ENaC, which plays an important role in fluid reabsorption by epithelium (for review, see Saint-Criq and Gray, 2017), is expressed at the apical membrane of endometrial epithelium (Chan et al., 2002; Enuka et al., 2012; Chinigarzadeh et al., 2015b). The protein abundance of ENaC is most abundant at diestrus, contributing to Na^+^ reabsorption by the endometrial epithelium (Chan et al., 2002; Enuka et al., 2012). Moreover, the expression of the apical ENaC and the basolateral Na^+^-K^+^ ATPase is stimulated by progesterone (Chinigarzadeh et al., 2015b). Consistent with the notion that NBCn1 is involved in ${\text{HCO}}_{3}^{-}$ reabsorption by the endometrial epithelium, our data show that the abundance of the uterine NBCn1 is highest at diestrus and its expression is stimulated by progesterone, but inhibited by β-estradiol. Finally, consistent with the up-regulation of NBCn1 and down-regulation of SLC26A4/A6 at diestrus, our *in vivo* perfusion study shows that the ${\text{HCO}}_{3}^{-}$ reabsorption activity of the endometrial epithelium at diestrus is significantly higher than that at estrus.

The secretion mode of the endometrial epithelium is likely stimulated by β-estradiol—the concentration of which is high during estrus. Indeed, the fluid secretion by the endometrial epithelium is enhanced by β-estradiol (Naftalin et al., 2002; Salleh et al., 2005). Consistent with the stimulatory effect of β-estradiol on the fluid secretion, the expression of CFTR in mouse uterus is most abundant at estrus (Chan et al., 2002). Moreover, the expressions of SLC26A4 and SLC26A6 are up-regulated by β-estradiol (He et al., 2010; Gholami et al., 2012) and data in the present study). The expression of NBCe1 is up-regulated by β-estradiol (Gholami et al., 2014). Finally, consistent with the expression profile of the presumable molecular machineries for ${\text{HCO}}_{3}^{-}$ secretion, our *in vivo* perfusion study on rat uterus shows that the ${\text{HCO}}_{3}^{-}$ secretion activity of the endometrial epithelium at estrus is significantly higher than that at diestrus.

Finally, it is worth to note that, the dual presence of Na^+^/${\text{HCO}}_{3}^{-}$ cotransporter NBCn1 (novel findings in the present study) and NHEs (reported previously) at the apical membrane of the endometrial epithelium are of general significance for understanding ${\text{HCO}}_{3}^{-}$ reabsorption and luminal acidification by epithelium. When stimulated, the submandibular salivary gland epithelium and pancreatic duct epithelium can secrete a large volume of fluid containing very high concentration of ${\text{HCO}}_{3}^{-}$ (Gennari and Weise, 2008). Interestingly, previous studies have shown that the epithelia in the submandibular salivary gland and pancreatic duct also express NBCn1 (termed as “NBC3”) and NHEs at the apical membrane contributing to reabsorb the luminal ${\text{HCO}}_{3}^{-}$ under resting condition (Lee et al., 1998, 2000; Luo et al., 2001; Park et al., 2002). In a more recent study, it is shown that the ${\text{HCO}}_{3}^{-}$ reabsorption in the proximal tubule epithelium involves the apical Na^+^/${\text{HCO}}_{3}^{-}$ cotransporter NBCn2 and NHE3 (Guo et al., 2017). Thus, the ${\text{HCO}}_{3}^{-}$ reabsorption (and therefore luminal acidification) in these tissues involves direct ${\text{HCO}}_{3}^{-}$-uptake via the apical NBCn1 and indirect ${\text{HCO}}_{3}^{-}$-reabsorption dependent on proton secretion via the apical NHEs. Our present study indicates that the endometrial epithelium employs similar strategies for the reabsorption of the luminal ${\text{HCO}}_{3}^{-}$ at diestrus.

In summary, in the present study, we provide evidence for the first time that the endometrial epithelium contains a ${\text{HCO}}_{3}^{-}$ reabsorption pathway involving the apical NBCn1. We propose that the acid-base homeostasis of the uterine fluid is regulated by the balance of the activities of the secretion vs. reabsorption for ${\text{HCO}}_{3}^{-}$, which is likely mediated by the coordinated interaction between the estrogen signaling and the progesterone signaling. During the estrus stage when β-estradiol is dominant, the molecular machineries for ${\text{HCO}}_{3}^{-}$ secretion are up-regulated whereas those for ${\text{HCO}}_{3}^{-}$ reabsorption are down-regulated to maintain the high concentration of ${\text{HCO}}_{3}^{-}$ in the uterine fluid, and vice versa during the diestrus stage when progesterone is dominant. Further studies are necessary to provide functional evidence for the involvement of NBCn1 and to address its specific contribution (relative to the apical NHEs) in the reabsorption of ${\text{HCO}}_{3}^{-}$ by the endometrial epithelium.

## Disclosures

We are grateful to Dr. Mark D. Parker at the State University of New York at Buffalo for critical editing of the manuscript. This work was supported by NSFC grants 31571201 (YL), 31371171 (L-MC), 81571388 (L-MC), and 31771294 (L-MC) as well as by grant 2016YXMS263 (YL) from the Fundamental Research Funds for the Central Universities of China.

## Author contributions

L-MC, YL, and Z-DX designed the study. Z-DX, Y-MG, and M-JR performed the experiments and data collection, JY, S-FW, and T-HX provided critical technical assistance. Z-DX, L-MC, and YL analyzed the data and wrote the paper. All authors approved the manuscript.

### Conflict of interest statement

The authors declare that the research was conducted in the absence of any commercial or financial relationships that could be construed as a potential conflict of interest.
