# Supplementary material for: The Balance of HCO3- Secretion vs. Reabsorption in the Endometrial Epithelium Regulates Uterine Fluid pH
Source: Front Physiol. 2018 Jan 25;9:12. doi: 10.3389/fphys.2018.00012 (PMC5788990; doi:10.3389/fphys.2018.00012)
Supplement: Supplementary file 1 [file DataSheet1.PDF]

Supplementary Material

The balance of machineries for secretion versus reabsorption of HCO_3^- in endometrium controls acid-base homeostasis in uterine fluid

Zhang-Dong Xie[1], Yi-Min Guo[1], Mei-Juan Ren[1], Jichun Yang[2], Shao-Fang Wang[3], Tong-Hui Xu[3], Li-Ming Chen[1]*, Ying Liu[1]*

[1]Department of Biophysics and Molecular Physiology, Key Laboratory of Molecular Biophysics of Ministry of Education, School of Life Science & Technology, Huazhong University of Science & Technology, Wuhan, Hubei 430074, China

[2]Department of Physiology and Pathophysiology, School of Basic Medical Sciences, Peking University Health Science Center, Beijing 100091, China

[3]Britton Chance Center for Biomedical Photonics, Wuhan National Laboratory for Optoelectronics, Huazhong University of Science and Technology, Wuhan 430074, China

Validation of NBCn1 antibody

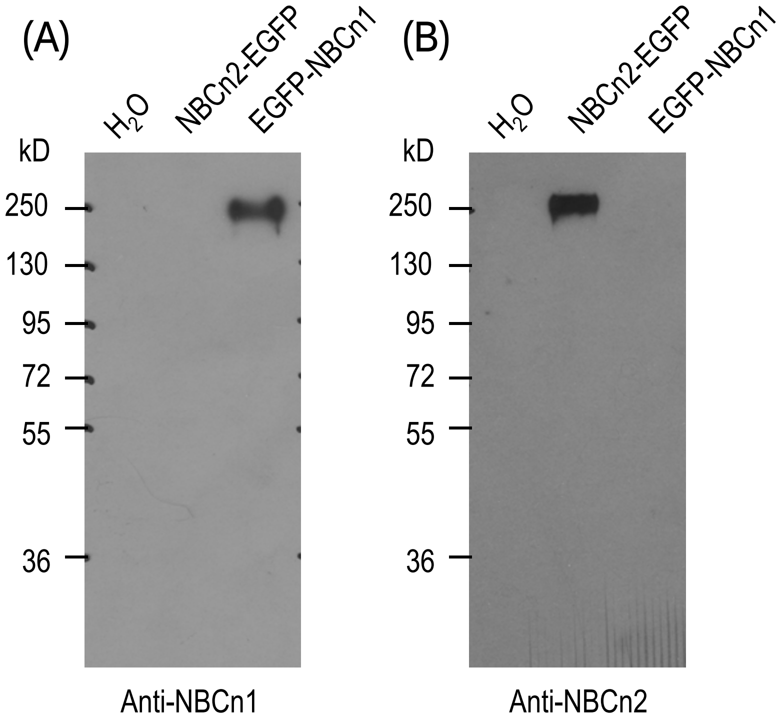

Supplementary Figure S1. Anti-NBCn1 specifically recognizes NBCn1 and does not cross react with NBCn2. cRNAs encoding mouse NBCn1 (SLC4A7) tagged with EGFP at amino terminus or rat NBCn2 (SLC4A10) tagged with EGFP at carboxyl terminus were injected into *Xenopus* oocytes. Control oocytes were injected with H_2O. Membrane proteins were prepared from the oocytes for western blotting analysis. Anti-NBCn1 antibody specifically recognizes NBCn1, but not NBCn2 (panel A), whereas anti-NBCn2 specifically recognizes NBCn2, but not NBCn1 (panel B). No band was detected in control oocytes injected with H_2O by either anti-NBCn1 or anti-NBCn2.

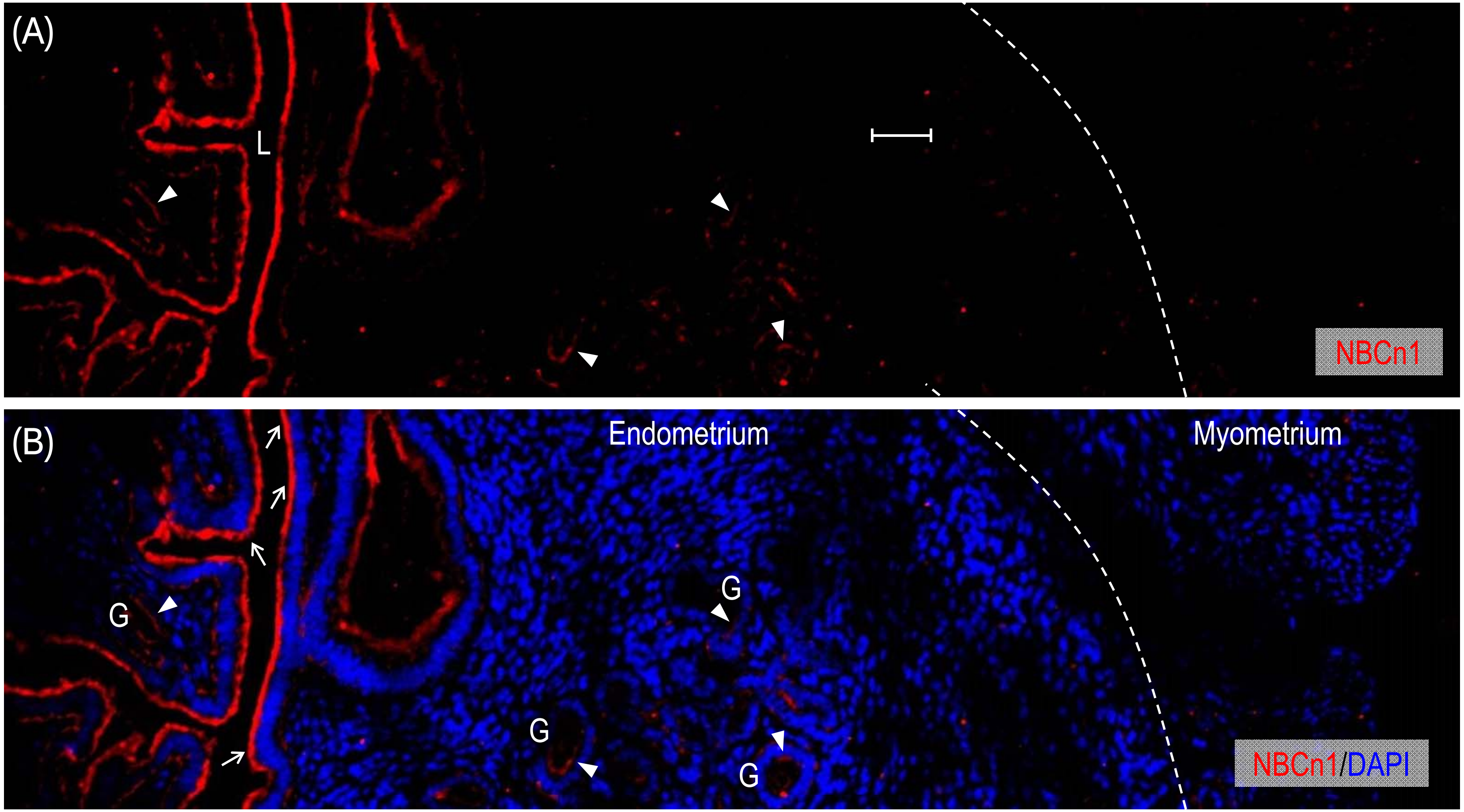

Supplementary Figure S2. Overview of indirect immunofluorescence of NBCn1 (A) and merge with DAPI (B) in a section of mouse uterus. The dashed lines indicate the demarcation between the myometrium layer and the endometrium layer. DAPI: 4,6-diamidino-2-phenylindole dihydrochloride; L: uterus lumen; G: grandular duct. Scale bar: 40 μm. Arrows indicate the expression of NBCn1 at the apical membrane of endometrial epithelia. Arrow heads indicate the expression of NBCn1 at the apical membrane of glandular epithelia.

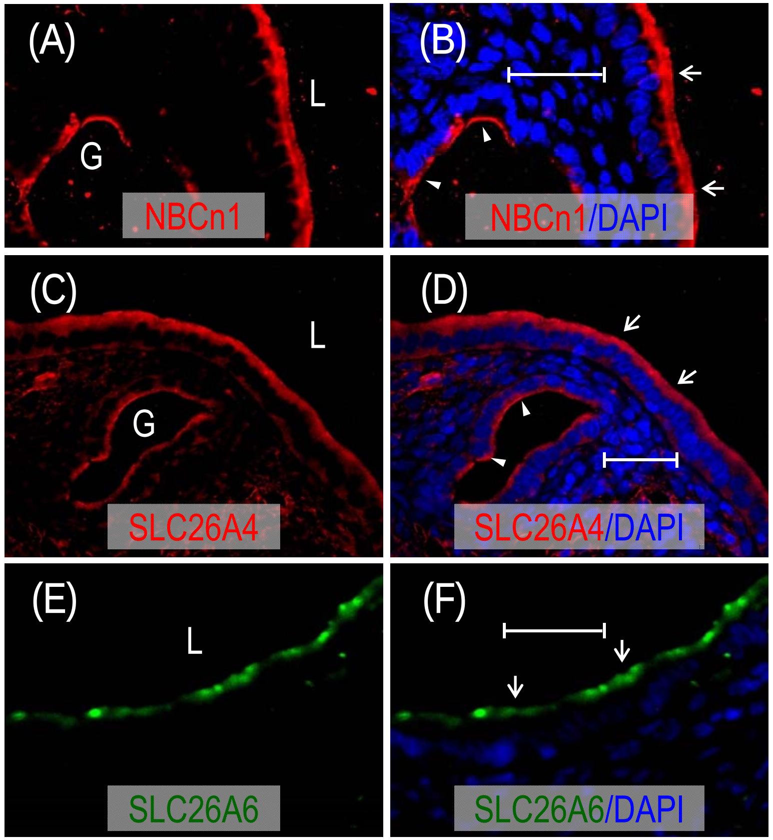

Supplementary Figure S3. Indirect immunofluorescence of NBCn1, SLC26A4, and SLC26A6 in sections of rat uterus. (**A-B**) Localization of NBCn1 in rat uterus. (**C-D**) Localization of SLC26A4 in rat uterus. (**E-F**) Localization of SLC26A6 in rat uterus. In these experiments, the rat was not intentionally examined to determine the estrous stage when sacrificed for uterus collection. L: uterus lumen; G: glandular duct. Arrows indicate the apical membrane of the endometrial epithelium. Arrowheads indicate the apical membrane of the glandular epithelium. Scale bars: 40 μm. Results are representative of 3 independent experiments.

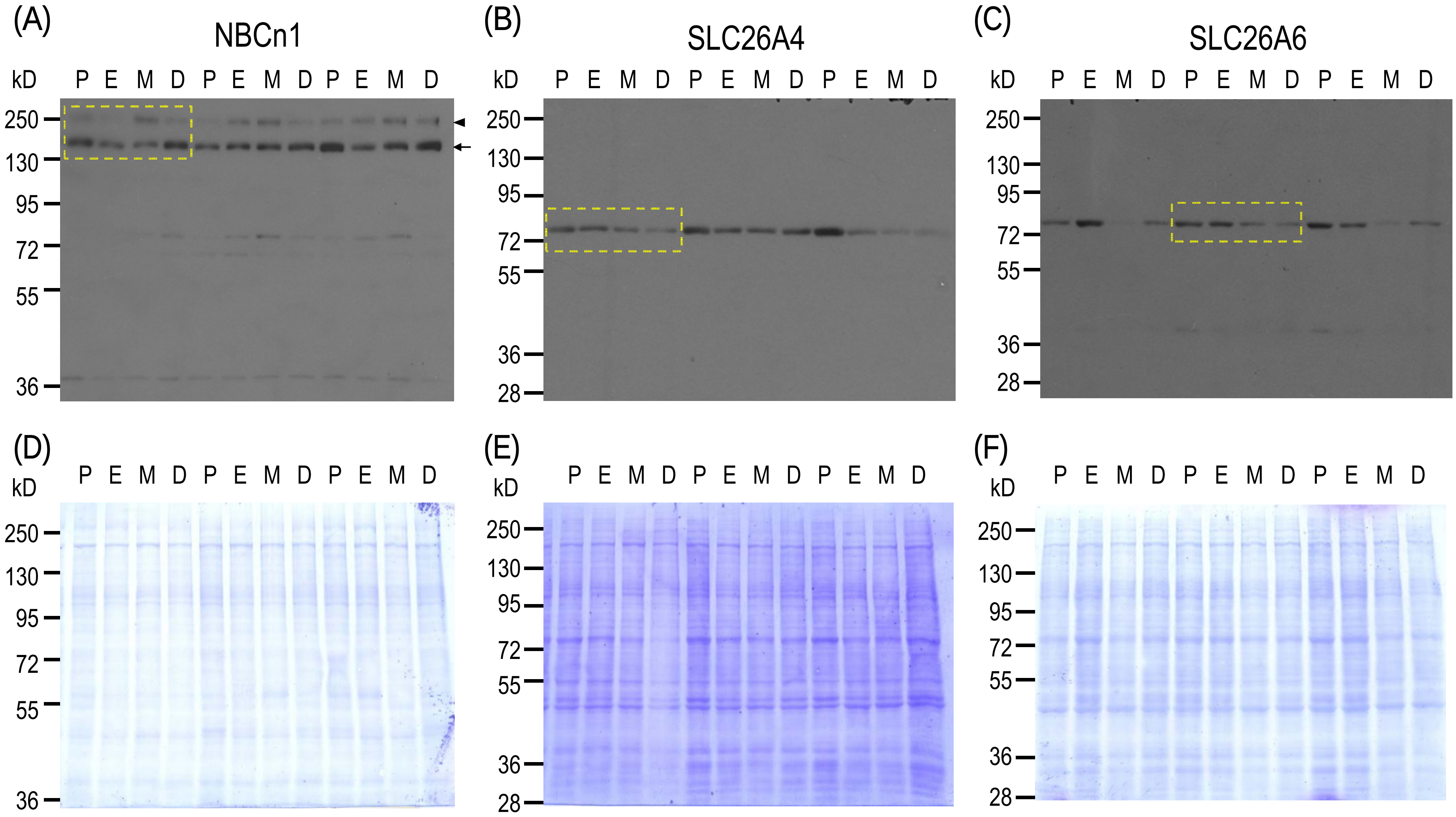

Supplementary Figure S4. See next page for figure legends.

Supplementary Figure S4. Expression of HCO_3^- transporters in mouse uteri at different estrous stages. (A) NBCn1. (B) SLC26A4. (C) SLC26A6. (D−F) Coomassie Brilliant Blue (CBB) staining of the corresponding blots shown in panels A−C. Crude membrane preparations were prepared from the uterus of each individual mouse, and protein concentration was quantitated as described in Method of the main text. Such membrane preparations were diluted with H_2O, mixed with SDS-sample buffer in such a manner that the concentration of total proteins in each of the final samples was identical. Equal volume of such final samples was then loaded onto the gel for SDS-PAGE and blotted onto a PVDF membrane for western blotting analysis. The dashed-line boxes in panels A−C indicate the regions shown in Figure 4A in the main text. The arrow in panel A indicates the presumable monomer of glycosylated NBCn1, whereas the arrowhead indicates the presumable dimer of glycosylated NBCn1. CBB staining shows that the loading is basically equal in each lane on the same blot. The variation in the width of lanes (e.g., the first lane "D" from left) was presumably due to the difference in the salt concentrations of the final samples for SDS-PAGE resulting from different dilutions with H_2O of the original crude membrane preparations. P: proestrus. E: estrus. M: metestrus. D: diestrus.

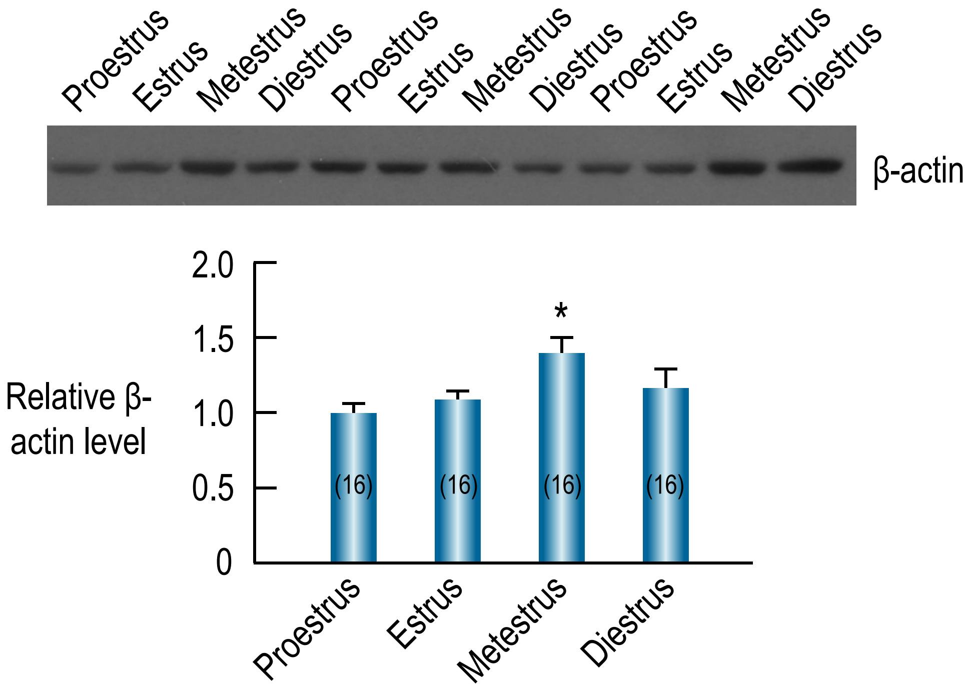

Supplementary Figure S5. Expression of β-actin in membrane preparations of mouse uteri at different estrous stages. The data for β-actin were obtained from the same batch of blots used for data collection for Figure 3/S4. The relative abundances of β-actin at proestrus, estrus, and diestrus are not significantly different from each other, but are significantly lower than the β-actin level at metestrus, suggesting that the expression of β-actin is not well conserved throughout the estrous cycle. One-way ANOVA followed by Fisher's post-hoc multiple comparisons was performed for statistical analysis. * $p<0.05$.

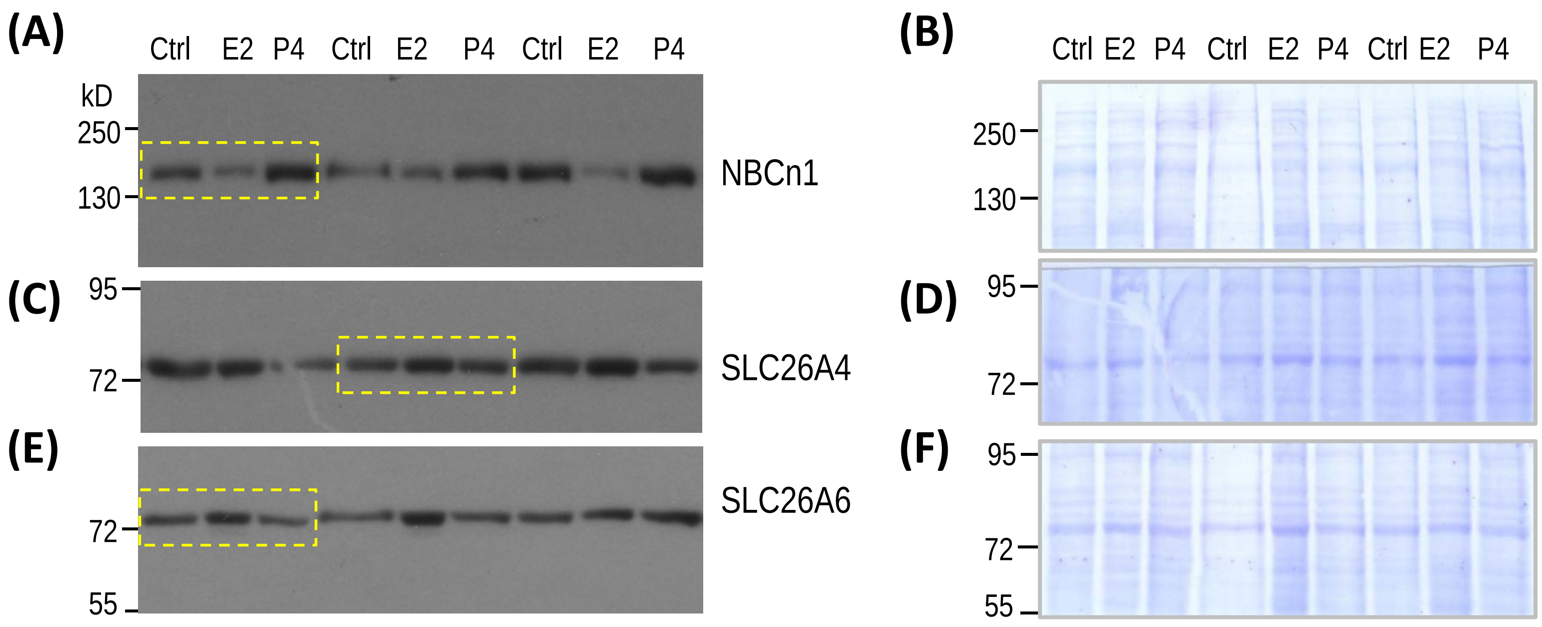

Supplementary Figure S6. Effect of estradiol (E2) and progesterone (P4) on expression of HCO_3^- transporters in the uteri of ovariectomized mice. (A, B) Representative western blotting of NBCn1 and coomassie staining of the blot. (C, D) Representative western blotting of SLC26A4 and coomassie staining of the blot. (E, F) Representative western blotting of SLC26A6 and coomassie staining of the blot. For western blotting analysis, equal amount of total membrane proteins were loaded into each lane, using a similar approach to that described in the legend to Supplementary Figure S3. The dashed-line boxes in panels A, C, and E indicate the portions shown in Figure 6 in the main text.

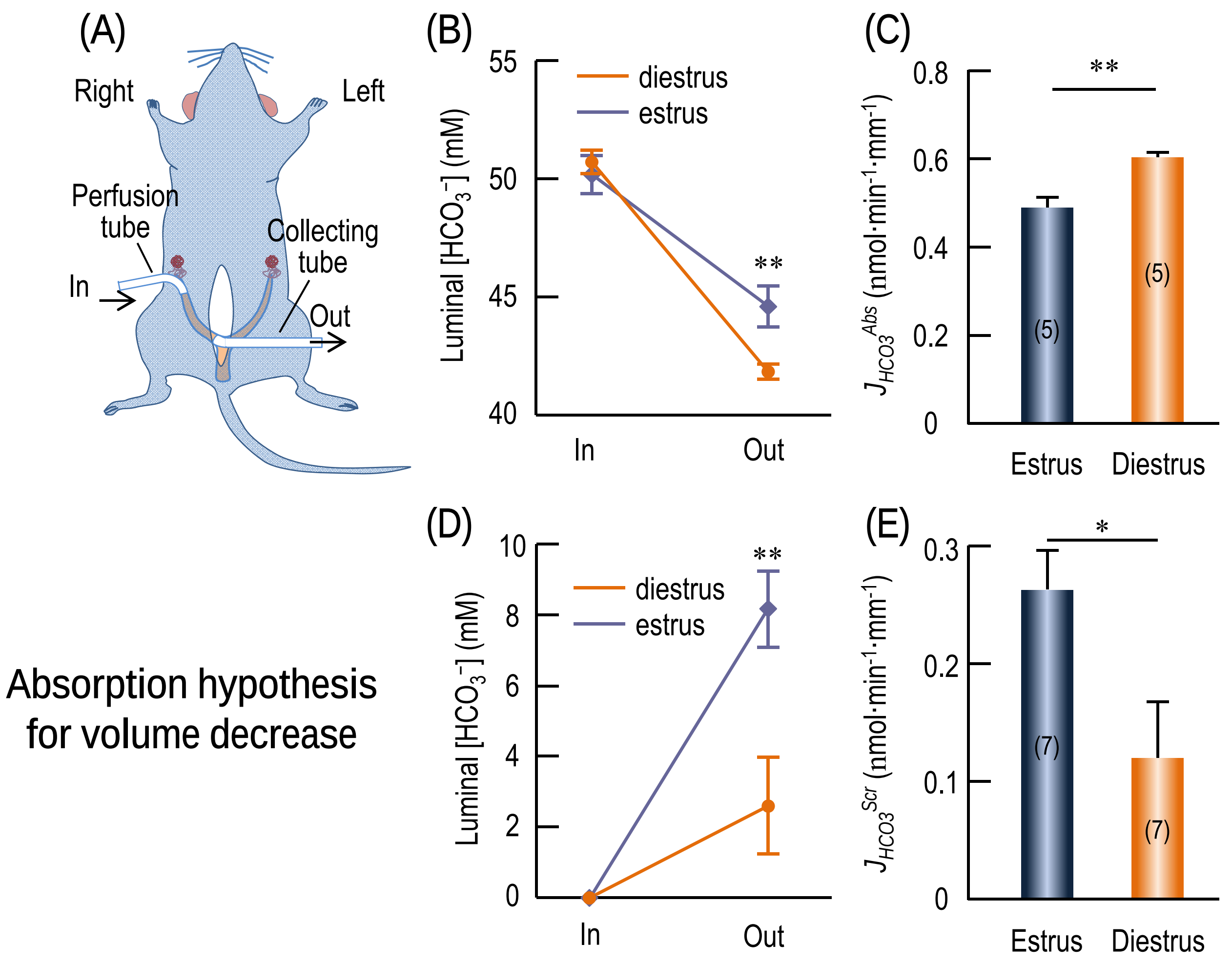

Supplementary Figure S7. *In vivo* perfusion study showing HCO_3^- transport by endometrial epithelium of rat uterus. Panels **A**, **B**, and **D** are the same as Figure 7A, B, and D in the main text. Panels **C** and **E** are calculated based upon the absorption hypothesis. For details, see text in the main text. * p<0.05; ** p<0.01; *** p<0.001.

HCO_3^- flux via the endometrial epithelium

The HCO_3^- absorption in our perfusion with 5% CO_2/50 mM HCO_3^- solution is unlikely to be mediated by the apical anion exchangers such as SLC26A4 and SLC26A6. SLC26A4 and SLC26A6 are reported to have a stoichiometry (Cl^- to HCO_3^-) of 1:1 and 1:2, respectively (Shcheynikov *et al.*, 2006; Shcheynikov *et al.*, 2008). Therefore, thermodynamically, these transporters would certainly mediate HCO_3^- secretion under the experimental condition. Note that, the HCO_3^- secretion by the anion exchangers in concert with CFTR could make up a luminal [HCO_3^-] to as high as 140 mM (Lee *et al.*, 2012). Instead, the HCO_3^- absorption could be explained by the apical NBCn1 which mediates HCO_3^- uptake driven by the inwardly-directed electrochemical gradient of Na^+. Alternatively, the HCO_3^- absorption could also be explained by a mechanism dependent on proton secretion mediated by the apical Na^+-H^+ exchangers (NHEs; see discussion on Figure 9A in the maintext).

In our perfusion with nominally HCO_3^--free solution containing 143 mM of Cl^-, the apical anion exchangers such as SLC26A4 and SLC26A6 would mediate HCO_3^- efflux only due to the inward electrochemical gradient of Cl^- and outward electrochemical gradient of HCO_3^-. In theory, the apical NBCn1 would mediate HCO_3^- efflux at the right beginning of the perfusion because the initial outward electrochemical gradient of HCO_3^- was infinitely large. However, the HCO_3^- efflux via NBCn1 would soon be reversed due to the accumulation of extracellular HCO_3^-. Suppose that the endometrial epithelium has typical intracellular ion concentrations, e.g., $[HCO_3^-]_i$ = 12 mM and $[Na^+]_i$ = 12 mM. Note that $[Na^+]_o$ = 126 mM (the luminal perfusate). A simple calculation shows that the HCO_3^- flux via NBCn1, which has an apparent stoichiometry of 1 Na^+ : 1 HCO_3^-, would become inward when the extracellular [HCO_3^-] is higher than 1.1 mM ($12\times12/126 \approx 1.1$ mM) (Chen *et al.*, 2016). Note that, the final [HCO_3^-] in the collected effluent fluid was ~8 mM for estrus and ~2.7 mM for diestrus (Figure 8B and 8D in the maintext). Thus, the HCO_3^- secretion by the endometrial epithelium would be largely attributable to the anion

exchangers such as SLC26A4 and SLC26A6, although one cannot completely rule out a contribution by NBCn1.

Reference

Chen, L.M., Liu, M., and Liu, Y. (2016). Electrophysiology principles of Na^+/HCO_3^- cotransporters. *Acta Physiologica Sinica*. 68, 323-334.

Lee, M.G., Ohana, E., Park, H.W., Yang, D., and Muallem, S. (2012). Molecular mechanism of pancreatic and salivary gland fluid and HCO_3^- secretion. *Physiol Rev*. 92, 39-74.

Shcheynikov, N., Wang, Y., Park, M., Ko, S.B., Dorwart, M., Naruse, S. *et al.* (2006). Coupling modes and stoichiometry of Cl^-/HCO_3^- exchange by slc26a3 and slc26a6. *J.Gen.Physiol*. 127, 511-524.

Shcheynikov, N., Yang, D., Wang, Y., Zeng, W., Karniski, L.P., So, I.*et al.* (2008). The Slc26a4 transporter functions as an electroneutral $Cl^-/I^-/HCO_3^-$ exchanger: role of Slc26a4 and Slc26a6 in I^- and HCO_3^- secretion and in regulation of CFTR in the parotid duct. *J. Physiol.* 586, 3813-3824.
